# Supplementary material for: Cholera outbreak in Forcibly Displaced Myanmar National (FDMN) from a small population segment in Cox’s Bazar, Bangladesh, 2019
Source: PLoS Negl Trop Dis. 2021 Sep 22;15(9):e0009618. doi: 10.1371/journal.pntd.0009618 (PMC8457470; doi:10.1371/journal.pntd.0009618)
Supplement: Attachment — Submitted filename: PNTD-D-21-00136_R1.pdf [file pntd.0009618.s007.pdf]

PLOS Neglected Tropical Diseases

Cholera outbreak in Forcibly Displaced Myanmar National (FDMN) from a small population segment in Cox's Bazar, Bangladesh, 2019

--Manuscript Draft--

**Manuscript Number:**	PNTD-D-21-00136R1
**Full Title:**	Cholera outbreak in Forcibly Displaced Myanmar National (FDMN) from a small population segment in Cox's Bazar, Bangladesh, 2019
**Short Title:**	A small Cholera outbreak in Forcibly Displaced Myanmar National
**Article Type:**	Research Article
**Keywords:**	Cholera, under-five children, displaced population, emergency and crisis settings, case management
**Abstract:**	Background: Bangladesh experienced a sudden, large influx of forcibly displaced persons from Myanmar in August 2017. A cholera outbreak occurred in the displaced population during September-December 2019. This study aims to describe the epidemiologic characteristics of cholera patients who were hospitalized in diarrhea treatment centers (DTCs) and sought care from settlements of Forcibly Displaced Myanmar Nationals (FDMN) as well as host country nationals during the cholera outbreak.<br>Methods : Diarrhea Treatment Center (DTC) based surveillance was carried out among the FDMN and host population in Teknaf and Leda DTCs hospitalized for cholera during September-December 2019.<br>Results: During the study period, 147 individuals with cholera were hospitalized. The majority, 72% of patients reported to Leda DTC. Nearly 65% sought care from FDMN settlements. About 47% of the cholera individuals were children less than 5 years old and 42% were aged 15 years and more. Half of the cholera patients were females. FDMN often reported from Camp # 26 (45%), followed by Camp # 24 (36%), and Camp # 27 (12%). Eighty-two percent of the cholera patients reported watery diarrhea. Some or severe dehydration was observed in 65% of cholera individuals. Eighty-one percent of people with cholera received pre-packaged ORS at home. About 88% of FDMN cholera patients reported consumption of public tap water. Pit latrine without water seal was often used by FDMN cholera individuals (78%).<br>Conclusion: Vigilance for cholera patients by routine surveillance, preparedness, and response readiness for surges and oral cholera vaccination campaigns can alleviate the threats of cholera.
**Additional Information:**	
**Question**	**Response**
**Financial Disclosure**<br><br>Enter a financial disclosure statement that describes the sources of funding for the work included in this submission. Review the submission guidelines for detailed requirements. View published research articles from *PLOS NTDs* for specific examples.<br><br>This statement is required for submission and **will appear in the published article** if the submission is accepted. Please make sure it is accurate.	Dr. ASG Faruque<br>Name Grant Number:GR-01875<br>UNICEF Bangladesh<br>The funders had no role in study design, data collection and analysis, decision to publish, or preparation of the manuscript.

**Unfunded studies**<br><br>Enter: *The author(s) received no specific funding for this work.*<br><br>**Funded studies**<br><br>Enter a statement with the following details:<br>• Initials of the authors who received each award<br>• Grant numbers awarded to each author<br>• The full name of each funder<br>• URL of each funder website<br>• Did the sponsors or funders play any role in the study design, data collection and analysis, decision to publish, or preparation of the manuscript?<br>• **NO** - Include this sentence at the end of your statement: *The funders had no role in study design, data collection and analysis, decision to publish, or preparation of the manuscript.*<br>• **YES** - Specify the role(s) played.<br><br>* typeset	
**Competing Interests**<br><br>Use the instructions below to enter a competing interest statement for this submission. On behalf of all authors, disclose any competing interests that could be perceived to bias this work—acknowledging all financial support and any other relevant financial or non-financial competing interests.<br><br>This statement **will appear in the published article** if the submission is accepted. Please make sure it is accurate. View published research articles from *PLOS NTDs* for specific examples.	The authors have declared that no competing interests exist.

**NO authors have competing interests**<br><br>Enter: *The authors have declared that no competing interests exist.*<br><br>**Authors with competing interests**<br><br>Enter competing interest details beginning with this statement:<br><br>*I have read the journal's policy and the authors of this manuscript have the following competing interests: [insert competing interests here]*<br><br>* typeset<br><br>This statement is required for submission and **will appear in the published article** if the submission is accepted. Please make sure it is accurate and that any funding sources listed in your Funding Information later in the submission form are also declared in your Financial Disclosure statement.	
**Data Availability**<br><br>Authors are required to make all data underlying the findings described fully available, without restriction, and from the time of publication. PLOS allows rare exceptions to address legal and ethical concerns. See the PLOS Data Policy and FAQ for detailed information.	No - some restrictions will apply

Powered by Editorial Manager® and ProduXion Manager® from Aries Systems Corporation

A Data Availability Statement describing where the data can be found is required at submission. Your answers to this question constitute the Data Availability Statement and **will be published in the article**, if accepted.<br><br>**Important:** Stating 'data available on request from the author' is not sufficient. If your data are only available upon request, select 'No' for the first question and explain your exceptional situation in the text box.<br><br>Do the authors confirm that all data underlying the findings described in their manuscript are fully available without restriction?	
**Describe where the data may be found in full sentences. If you are copying our sample text, replace any instances of XXX with the appropriate details.**<br><br>• If the data are **held or will be held in a public repository**, include URLs, accession numbers or DOIs. If this information will only be available after acceptance, indicate this by ticking the box below. For example: *All XXX files are available from the XXX database (accession number(s) XXX, XXX.*).<br>• If the data are all contained **within the manuscript and/or Supporting Information files**, enter the following: *All relevant data are within the manuscript and its Supporting Information files.*<br>• If neither of these applies but you are able to provide **details of access elsewhere**, with or without limitations, please do so. For example:<br><br>*Data cannot be shared publicly because*	It is prudent to mention that the data of this manuscript has been obtained from a large study with a huge data set that deals with a number of objectives. The submitted manuscript deals with one of the objectives of that data set. This data set contains some personal information of the study patients (such as name, admission date, month, area of residence). However, during taking the consent from the patients, it has been ensured that the personal information of them will not be disclosed, but, the study results will be published. Thus, the availability of this whole data set in the manuscript, the supplemental files, or a public repository will open all the personal information of the patients those should not be disclosed; additionally this will disclose other important information those are yet to be published. Thus, the policy of our centre (icddr,b) is that we should not make the availability of whole data set in the manuscript, the supplemental files, or a public repository. However, part of data set related to this manuscript is available upon request and readers may contact with Ms. Armana Ahmed (aahmed@icddrb.org) to the Research Administration & Strategy of icddr,b to request the data (http://www.icddrb.org/)."

of [XXX]. Data are available from the XXX Institutional Data Access / Ethics Committee (contact via XXX) for researchers who meet the criteria for access to confidential data.<br><br>*The data underlying the results presented in the study are available from (include the name of the third party and contact information or URL).*<br>• This text is appropriate if the data are owned by a third party and authors do not have permission to share the data.<br><br>* typeset	
Additional data availability information:	Tick here if the URLs/accession numbers/DOIs will be available only after acceptance of the manuscript for publication so that we can ensure their inclusion before publication.

Powered by Editorial Manager® and ProduXion Manager® from Aries Systems Corporation

Manuscript

Click here to access/download;Manuscript;Plos NTD_Coxs_Bazar_ASGF. revised.25.1.2021.doc

Cholera outbreak in Forcibly Displaced Myanmar National (FDMN) from a small population segment in Cox's Bazar, Bangladesh, 2019

ASG Faruque[1¶*], Azharul Islam Khan[1¶], Baitun Nahar[1], SM Rafiqul Islam[1], M Nasif Hossain[1], Syed Asif Abdullah[1], Soroar Hossain Khan[1], Md. Sabbir Hossain[2], Feroz Hayat Khan[2], Mukesh Prajapati[2], Yulia Widiati[3], ASM Mainul Hasan[3], Minjoon Kim[4], Jennie Musto[2], Maya Vandenent[4], John David Clemens[1,5], Tahmeed Ahmed[1]

[1]Nutrition and Clinical Services Division, icddr,b, Dhaka 1212, Bangladesh

[2]World Health Organization, Cox's Bazar, Bangladesh

[3]UNICEF Bangladesh, Cox's Bazar Field Office

[4]UNICEF Bangladesh Country Office

[5]UCLA Fielding School of Public Health, USA

¶ These authors Contributed equally to this work as joint first authors

*ASG Faruque

Email: gfaruque@icddrb.org

Abstract

Objective: Bangladesh experienced a sudden, large influx of forcibly displaced persons from Myanmar in August 2017. A cholera outbreak occurred in the displaced population during September-December 2019. This study aims to describe the characteristics of cholera cases, their care-seeking pattern, camp-wise distribution of Forcibly Displaced Myanmar National (FDMN) cases, sources of drinking water, toilet use pattern, oral cholera vaccine (OCV) status, and share the experiences from effective interventions to prevent a cholera outbreak.

Methods*:* Diarrhea Treatment Center (DTC) based surveillance was carried out among the FDMN in Teknaf and Leda DTCs for cholera during September-December 2019.

Results: During the study period, 147 cases of cholera were hospitalized. The majority, 72% of cases reported to Leda DTC. Nearly 65% sought care from FDMN settlements. About 47% of the cholera cases were children less than 5 years old and 42% were aged 15 years and more. Half of the cholera cases were females. FDMN often reported from Camp # 26 (45%), followed by Camp # 24 (36%), and Camp # 27 (12%). Eighty-two percent of the cases reported watery diarrhea. Some or severe dehydration was observed in 65% of cholera cases. Eighty-one percent of cases received pre-packaged ORS at home. About 88% of FDMN cholera cases reported consumption of public tap water. Pit latrine without water seal was often used by FDMN cholera cases (78%).

Conclusion: Vigilance for cholera cases by routine surveillance, preparedness, and response readiness for surges and OCV campaigns can alleviate the threats of cholera.

Keywords. Cholera, under-five children, displaced population, emergency and crisis settings, case management

Author Summary

Bangladesh observed an unexpected, large-scale arrival of forcibly displaced individuals from Myanmar in August 2017. The Bangladesh Government, UN agencies, and international and national non-governmental organizations responded immediately with extensive humanitarian response. However, because of the exceptionally large size of the displaced population, and the presence of inadequate lifesaving infrastructures of sanitation, threats of acute watery diarrhea, cholera, and shigellosis outbreaks were prevailing. The Government of Bangladesh as lead, with technical support from icddr,b collaborating with international agencies undertook a massive oral cholera vaccination (OCV) campaign immediately as a pre-emptive measure to alleviate threats of cholera outbreak. Despite that mass OCV campaign, threats of cholera outbreak among Forcibly Displaced Myanmar Nationals were existing due to new arrivals of the displaced population with compromised host susceptibility, frequent visits to settlements by Bangladesh nationals without exposure to OCV and, the declining vaccine immunity among OCV recipients as well as an increasing number of cohort children without any exposure to OCV. The population faced a cholera outbreak during September-December 2019. This study aims to describe the characteristics of cholera cases from that outbreak, their care-seeking pattern, camp-wise distribution, source of drinking water, sanitation facility, OCV status, and share the experiences from effective interventions to prevent a cholera outbreak. Vigilance for cholera cases by routine surveillance, preparedness, and response readiness for surges and OCV campaigns can alleviate the threats of cholera.

Introduction

In August 2017, Bangladesh witnessed a sudden influx of an estimated 745,000 Forcibly Displaced Myanmar Nationals (FDMN) including more than 400,000 children within 17 weeks from neighboring Rakhine state in Myanmar who settled in the Cox's Bazar district situated in the south-east of the country. Their journey to Bangladesh was hazardous with limited access to food and water, often had diverse injuries and illnesses, crossed through jungles and mountainous terrain and finally, most of them had a boat ride to cross the Naf river while some confronted risky sea voyage across the Bay of Bengal. The Bangladesh Government, UN agencies, and a large number of international and national non-governmental organizations (NGOs) reacted immediately with a large-scale humanitarian response. Camps were established quickly but soon humanitarian agencies started struggling to meet the exorbitant demand for assistance and supplies. The displaced population urgently needed critical supplies like medicine, clean water, food, and shelter with special attention to children, women, the elderly, and disabled individuals. Many of the hurriedly built camps were vulnerable to monsoon flooding and storm surges. Those families who started living in hillsides were prone to landslides. Latrines and shallow and deep tube wells were constructed to protect against public health issues and ensure access to clean water. However, because of the arrival of a large number of displaced populations and the presence of insufficient lifesaving infrastructures of sanitation, like latrines and water points, the environment soon became a breeding ground for waterborne diseases including acute watery diarrhea, cholera, and shigellosis. These risks were further heightened by high population density in camps and an excess number of severely malnourished children who yield even more quickly to preventable and treatable diseases as well as outbreaks of acute watery diarrhea (AWD), cholera, and shigellosis [1–7].

Almost immediately, following the huge influx and settlement of these displaced populations, UNICEF-Bangladesh and icddr,b jointly conducted a brief field assessment in Ukhia and Teknaf sub-districts of Cox's Bazar. The assessment anticipated potential threats of diarrheal disease outbreaks including cholera and shigellosis, and strategies were immediately set to initiate mitigation measures. A partnership between icddr,b, and UNICEF under the umbrella of Health Sector targeted (i) training doctors, nurses, and community health workers of the government and NGO run facilities serving FDMN in the settlements as well as host population living in the neighborhood housing; (ii) managing cases of dehydrating diarrheal episodes and associated malnutrition through a network of five diarrhea treatment centers (DTCs); and carrying out DTC based diarrheal disease surveillance as it is known to be critical for early detection of outbreaks. Activities of the diarrheal disease surveillance team included data collection, a one-step rapid diagnostic test for the presence of *Vibrio cholerae* in stool specimen of hospitalized patients, and microbial tests to detect common enteric pathogens including *Vibrio cholerae* by submitting fecal specimens directly as well as after inoculation into Cary-Blair Transport Medium to the Clinical Microbiology Laboratory of icddr,b in Dhaka, Bangladesh.

The Government of Bangladesh as lead, with technical support from icddr,b collaborating with international agencies, and international and national NGOs under the wider platform of Health Sector, undertook a massive oral cholera vaccination (OCV) campaign immediately as a pre-emptive measure to alleviate threats of cholera outbreaks [8–10]. Despite that mass oral cholera vaccination campaign, threats of cholera outbreaks among FDMN were existing due to new arrivals of the displaced population with compromised host susceptibility, frequent visits to settlements by Bangladesh nationals living in the neighboring community without exposure to

OCV and, the decay of vaccine immunity in among OCV recipients as well as an increasing number of cohort children without any exposure to OCV. Preparedness for combating surges and vigilance for cholera cases were the most important public health priorities because of prevailing threats of cholera in both the host and displaced population [11,12].

icddr,b, and UNICEF jointly organized a dissemination session for the local stakeholders on their activities for the FDMN living in the settlements in March 2019. Between September and December 2019, there have been 147 cases of culture-confirmed cholera who presented and subsequently hospitalized with acute dehydrating diarrhea episodes in Leda and Teknaf DTCs. Thus, it became essential to share this cholera outbreak control experience with policymakers, public health teams, program managers, academia, and wider stakeholders acquired from a perspective of sufficiently prepared, field-based, and well-tailored strategy in an emergency and crisis setting. Such experience sharing is not a common and widespread phenomenon, particularly in humanitarian emergencies. An update of this kind is likely to enable stakeholders to undertake necessary preparedness to prevent cholera outbreaks from occurring and to respond successfully when the outbreaks have occurred.

This paper aims to (i) describe the characteristics of cholera cases including that of FDMN care seekers, their reporting pattern to DTCs, camp-wise distribution, and OCV status, (ii) compare drinking water sources and toilet use pattern between FDMN and host community cholera cases, (iii) describe comparative clinical and demographic characteristics between cholera cases who sought care from Cox's Bazar DTCs, and Dhaka Hospital of icddr,b during the same period, and (iv) share the experiences that were obtained from this cholera outbreak that occurred in a small

segment of the FDMN living in settlements of Cox's Bazar, Bangladesh.

Methods

Setting and study population

This was a DTC based cross-sectional diarrheal disease surveillance for FDMN and host community individuals hospitalized in DTCs.

In late September 2019, two cholera cases for the first time after two years of the arrival of FDMN were detected in Teknaf DTC which is run by icddr,b. They sought care from settlements (one from Camp # 25 and the other from Camp # 26). Such an incident was reported immediately to the Epidemiology Team Lead and Early Warning, Alert and Response System (EWARS) of WHO-Cox's Bazar, as well as UNICEF-Cox's Bazar. The next day, Cox's Bazar Health Sector's Joint Assessment Team (JAT) consisting of Health and WASH Sector partners investigated the hotspots and affected camps. The JAT reported worsening hygiene practices and sanitary conditions as a result of an acute shortage of safe drinking water, and the use of stagnant contaminated water for domestic purposes. Several recommendations were made on that day including hygiene promotion in the hot spots, desludging of latrines as soon as possible, distribution of water purifying tablets, pre-packaged ORS, soap, and chlorine by the WASH Sector, and availability of a handwashing facility in the latrine areas. The stagnant contaminated pools of water were fenced to prevent access to it by people living in its surroundings. Urgent refresher training on risk assessment for health teams was recommended. Within 24 hours, one temporarily closed down DTC in Leda close by the affected settlements was reopened to serve the increasing number of AWD cases.

The Health Systems of Bangladesh Government joined WHO-Cox's Bazar in streamlining activities of EWARS. Such an exertion strengthened monitoring of the cases of AWD and cholera in the camps for early detection and response to outbreaks. Moreover, a meeting of the Directorate General of Health Services and leading international agencies responsible for emergency responses in Cox's Bazar was followed by the institution of immediate alleviation measures that included the supply of safe drinking water and improvement of the sanitation system. To ensure adequate clinical management of AWD cases following a standard management protocol, the existing network of DTCs was strengthened by UNICEF-Cox's Bazar. WHO and the Health Sector recommended that those cases presenting to the out-patient clinics with dehydrating diarrhea should be immediately referred to Diarrhea Treatment Centres (DTCs) run by icddr,b, or, if there were no DTCs nearby, to primary health care centers (PHCs) with isolation facilities. Leda DTC (14 beds) and Teknaf DTC (30 beds) located in the neighborhood of settlements remained open as usual round-the-clock. Six batches of the health workforce were immediately trained by icddr,b on the clinical management of AWD cases. Community health workers were also assigned by UNICEF-Cox's Bazar in outreach activities including promotion of good hygiene practices and combatting diarrhea episodes at the household level with the use of pre-packaged ORS as soon as there is the onset of these episodes [13–16].

Preparations and response readiness were undertaken for the acceleration of the existing cholera vaccination campaign as an increasing trend of dehydrating diarrhea cases in DTCs was revealed. As a result, the International Coordinating Group for Cholera Vaccine (ICG) Secretariat approved a request for additional 1.2 million doses of OCV. Ministry of Health and Family Welfare, Bangladesh playing the leading role with the support of WHO, UNICEF, and

other partners, the campaign started vaccinating those individuals living in the neighborhood host community but yet to receive any OCV. The OCV campaign (including operational cost) was funded by GAVI, the Vaccine Alliance. The vaccination operation aimed mostly to reach displaced children aged 12-59 months. In the host community, the campaign looked for any person aged 1 year or more, because approximately 80% of host community people residing near the settlements were never targeted to receive OCV in previous campaigns although they were equally vulnerable like the FDMN [13,14].

Routine enteric pathogen detection activities that included a collection of a single stool specimen (of at least 3 g) directly from the patients following hospitalization were ongoing. Soon after collection, a one-step rapid diagnostic test was performed by SD BIOLINE cholera antigen O1/O139 (44FK30) test kit, supplied by WHO-Cox's Bazar, which is an immunochromatographic test for the qualitative detection of *Vibrio cholerae* O1/O139 in human stool specimens (manufactured by STANDARD DIAGNOSTICS, INC located in Suwon city, Kyonggi province, Republic of Korea). To facilitate microbial culture to confirm the rapid diagnostic test results; the provisionally diagnosed specimens (the stool) of cholera cases were inoculated into Cary-Blair Transport Medium; and the medium was then sent as soon as possible to the Clinical Microbiology Laboratory, icddr,b, based in Dhaka, Bangladesh to isolate the colony as well as perform antibiotic susceptibility tests with immediate sharing of the results to the concerned DTC, Epidemiology Team Lead of WHO-Cox's Bazar and UNICEF-Cox's Bazar. Other non-positive by rapid diagnostic test specimens were submitted routinely once or twice a week [17–19].

In daily monitoring, evaluation, and reporting, icddr,b followed its expertise gathered from its hospital-based Diarrheal Disease Surveillance System (DDSS) which is in operation in icddr,b's urban Dhaka (since 1979), and rural Matlab (since 1999) facilities. The Diarrheal Disease Surveillance System (DDSS) at Dhaka Hospital enrolls a 2% systematic sample of patients reporting to the triage area. Patients seeking care from the Matlab Hospital who are residents of the Demographic Surveillance System (DSS) area are enrolled into the DDSS. Trained enumerators using structured questionnaires interview patients and/or their attendants to collect relevant information on socioeconomic and demographic profile, housing and settings of the adjacent environment, feeding practices particularly of infants and toddlers, and use of drugs and fluid therapy at home before reporting to the facility. Additional information that is recorded includes clinical features, anthropometric measurements, treatments received in the facilities, and the outcome of the patients. Microbiological assessments are performed to identify common diarrheal pathogens and document the microbial susceptibility pattern of the bacterial pathogens. The activity offers useful information to hospital clinicians in their clinical decision-making courses and empowers icddr,b to detect the emergence of new enteric pathogens and early recognition of outbreaks and their locations, thereby guiding the host government to take suitable preventive and control measures [17–19]. Ongoing data collection by trained research assistants entailed administering structured questionnaires, from all hospitalized patients in DTCs and/or their attendants to gather information such as presenting clinical features, socioeconomic and demographic contexts, water, sanitation and hygiene, housing and its surrounding environment, feeding practices, particularly of 0-35 months old, and use of drugs and pre-packaged ORS at home before coming to DTC continued round-the-clock.

Ethics statement

The data collection process of this study was part of the ongoing activities entitled: *Surveillance for etiologic agents, care-seeking behavior, the status of IYCF and WASH practices among patients or their caregivers from Rohingya refugees as well as host population in Cox's Bazar district attending icddr,b operated Diarrhea Treatment Centers* was approved by icddr,b's (International Centre for Diarrhoeal Disease Research, Bangladesh) IRB comprising Research Review Committee (RRC) and Ethical Review Committee (ERC). Voluntary written informed consent was obtained from the parent/guardian before starting the interviewing process.

Statistical analysis

Data were analyzed by STATA (StataCorp version 13) and analyses included descriptive methods. Variables were described using frequencies with percentages. Exposure categories were compared using Pearson χ^2 tests for categorical variables. Relevant data from the ongoing DDSS database of Dhaka Hospital were extracted for the period September-December, 2019 for a comparative analysis of clinical and demographic profiles of visiting culture-proven cholera cases between Cox's Bazar DTCs and Dhaka Hospital of icddr,b.

Results

Between September and December 2019, there were147 cases of culture-confirmed cholera who presented and were subsequently hospitalized with acute dehydrating diarrhea episodes in Leda and Teknaf DTCs. The majority, 72% of cases reported to Leda DTC. Nearly 65% of these cholera cases sought care from FDMN settlements. FDMN often reported to DTCs from Camp # 26 (45%), followed by Camp # 24 (36%), and Camp # 27 (12%). About 94% of the cholera cases

from the host community and 65% of the cholera cases from FDMN living in settlements did not receive any OCV before their onset of culture-proven cholera episodes (Table 1).

Table 1 Distribution of characteristics of culture-confirmed cholera patients (n=147) in Leda and Teknaf DTCs in Cox's Bazar settlements, September-December 2019

Variables name	n (%)
**Sought care from**	
Leda DTC	106 (72.1)
Teknaf	41 (27.9)
**Currently living in**	
Settlements	95 (64.6)
Host community	52 (35.4)
**From settlements**	
Camp # 26	43 (45.3)
Camp # 24	34 (35.8)
Camp # 27	11 (11.6)
Camp # 25	4 (4.2)
Camp # 15	2 (2.1)
Camp # 23	1 (1.1)
**Not exposed to OCV**	
FDMN	62 (65.3)
Host community individuals	49 (94.2)

DTC: Diarrhea treatment center; OCV: Oral cholera vaccine

The major sources of drinking water of the hospitalized displaced cholera cases were public tap installed in the settlements, deep tube-well, and shallow tube well. Use of public tap water was significantly more frequent in cholera cases from settlements than from the host community (88% vs. 10%; $p<0.001$). However, the use of deep tube well (6% vs. 21%; $p=0.005$) and shallow tube well (2% vs. 54%; $p<0.001$) water was significantly less common in the cholera cases from settlements. Nearly 78% of the displaced cholera cases used pit latrines without water seal as opposed to 44% of the cholera patients from the host community ($p<0.001$). However, the use of a pit latrine with a water seal was identical in both the groups (Table 2).

Table 2 Water source and toilet use by the culture-confirmed cholera patients in Leda and Teknaf DTCs in Cox's Bazar settlements, September-December 2019

Variables	FDMN *n=95* (%)	Host community *n=52* (%)	P-value
**Water source**			
Public tap	84 (88.4)	5 (9.6)	<0.001
Deep tube well	6 (6.3)	11 (21.2)	
Shallow tube well	2 (2.1)	28 (53.8)	
Others	3 (3.2)	8 (15.4)	
**Toilet use pattern**			
Pit latrine without water seal	74 (77.9)	23 (44.2)	<0.001
Pit latrine with water seal	21 (22.1)	12 (23.1)	
Others	0 (0.0)	17 (32.7)	

During September-December 2019, a total of 216 culture-confirmed cholera cases were hospitalized in icddr,b's Dhaka Hospital, and none had received OCV. During the same period, DTC logs reported the admission of 147 culture-proven cholera cases in Leda and Teknaf DTCs. Among these cholera cases, infants (p<0.001) and overall children <5 years old (p<0.001) presented more frequently to the DTCs (functioning to treat FDMN living in settlements as well as host community individuals) compared to cholera children presenting to Dhaka Hospital from Dhaka city and its suburbs (47% vs. 12%; p<0.001). However, for individuals aged 15 years and higher, more cholera patients reported to Dhaka Hospital as opposed to cholera cases living in settlements and seeking care from DTCs (76% vs. 42%; p<0.001) (Table 3). Significantly more female cholera cases visited DTCs as opposed to female cholera patients presenting to Dhaka Hospital (50% vs. 38%, p<0.043). Cholera cases in Dhaka Hospital more commonly presented with watery diarrhea than cholera patients of DTCs (100% vs. 82%, p<0.001), sought care more frequently with some or severe dehydration (98% vs. 65%, p<0.001), and had more access to ORS at home before seeking care (91% vs. 81%, p<0.010) (Table 3).

Table 3 Age stratified cholera cases in Dhaka Hospital and DTCs in Cox's Bazar settlements, September-December 2019

Variables	Dhaka hospital	DTCs in settlements	p-value

	n=216 (%)	n=147 (%)	
**Age (Year)**			
<1	3 (1.4)	14 (9.5)	<0.001
<5	25 (11.6)	69 (46.9)	
5-14	28 (13.0)	17 (11.6)	
15 and more	163 (75.5)	61 (41.5)	
**Range**	7 months – 74 years	3 months – 85 years	
**Female**	83 (38.4)	73 (49.7)	0.043
**Duration of diarrhea**			
<1 day	153 (70.8)	100 (68.0)	0.300
1-3 days	57 (26.4)	38 (25.9)	
4 days and more	6 (2.8)	9 (6.1)	
**Watery stool**	216 (100.0)	120 (81.6)	<0.001
**Some or severe dehydration**	211 (97.7)	95 (64.6)	<0.001
**Pre-packaged ORS us at home**	196 (90.7)	119 (81.0)	0.010

DTC: Diarrhea treatment center; ORS: Oral rehydration solution

Discussion

Humanitarian emergencies increase the risk of infectious disease transmission including cholera and shigellosis, and the prevalence of other health conditions such as severe undernutrition. An effective disease surveillance system is critical for early detection of disease outbreaks before any spread to other family members as well as individuals living in the neighborhood, unnecessarily costing lives and challenging the disease control efforts. Thus, our ongoing DTC based diarrheal disease surveillance system with timely laboratory back-up and immediate reporting was noteworthy in this emergency and crisis setting. The surveillance system was involved not only in collecting reliable data since the inception of the DTC network but also in reporting immediately to help significantly in anticipating and detecting early potential cholera outbreaks. Findings from surveillance system guided intervention strategies that lead to the timely undertaking of preventive measures and preparedness including training of health care staff and prepositioning of supplies and additional human resources. Additionally, surveillance data helped in identifying vulnerable populations living in high-risk areas who might have been benefitted from preventive OCV use. Thus, reliable epidemiological data was critical in the

efficient implementation of preventive as well as control measures. The present study observed that 94% of the host community individuals and two-third of the FDMN with laboratory-confirmed cholera were not exposed to OCV before getting hospitalized with AWD.

A recent experience from Bangladesh and India indicated that the protective efficacy of Shanchol OCV (produced in India) among those more than five years against cholera is 53-65%. The study mentioned the positive role of OCV as a pre-emptive measure in endemic settings, in natural or man-made disasters even in disruptive situations with a breakdown of WASH and absence of other disease control and public health measures [20]. WHO and Global Task Force for Cholera Control (GTFCC) recommend that a comprehensive multi-sectoral involvement is important for the successful elimination of cholera [21]. Mass OCV campaigns with high coverage are feasible even after the arrival of a large number of displaced populations in a distressed state in resource poor-settings like Bangladesh [8,9]. According to another study, OCV induced optimal immune responses in FDMN adults and children which were similar to that observed in Bangladesh's population of diverse age groups or individuals living in other cholera endemic countries [10].

In this study, we have explored the clinical, demographic, and hygienic practices of the displaced as well as the host population living in settlements and neighboring host communities. The findings of this study have public health implications and may be useful for the Health System of Government of Bangladesh for anticipation, preparedness, and implementation of preventive and mitigation measures in settings with public health threats such as endemic disease surges like cholera or it is breaking out into epidemic proportions. Additionally, vigilance for cause-specific

diarrhea surges in both the populations such as host and FDMN is critical. Several findings related to care-seeking from DTCs were noteworthy. Unlike Dhaka hospital, children living in settlements and host communities were more often hospitalized for culture-proven cholera episodes than their peers from Dhaka city and its suburbs. These observations underscore the need for OCV campaigns. Females aged 15 years and higher living in settlements were more often hospitalized with cholera than their peers seeking care from Dhaka Hospital. This may be due to the increased vulnerability of females living in settlements to cholera because of their higher compromised immunity or excess exposure to contaminated water and food during household activities. Excess reporting of male cholera cases in Dhaka Hospital may be due to increased mobility of male individuals as well as their frequent exposures to day-time unhygienic out-door street-side meals or snacks from vendors in the overcrowded megacity.

ORS use at home was significantly lower in the cholera cases seeking care from DTCs than those cholera cases living in Dhaka city and its suburbs. Likely explanations include less access to ORS packets at the household or community level because of less organized outreach activities to promote ORS at the household level in settlements. Alternatively, the displaced population was not optimally motivated to start ORS before coming to DTCs. Access to more safe water (chlorinated water supplied through taps installed) was observed in settlements mostly for FDMN as provided by international agencies and NGOs. However, their access to deep and shallow tube well water was less commonly observed compared to that of admissions from the host community. Cholera cases with significantly more frequent watery stool and with more frequent evidence of some or severe dehydration in Dhaka hospital could be due to more full-blown clinical features of cholera episodes which may be because of larger inoculum size that

may be ingested by those living in the more contaminated environment particularly in slums with gross lack of water and sanitation services as well as worsening hygienic practices in Dhaka city and its suburbs.

In Sudan among the displaced populations, the risks for cholera were considerably higher among children less than five years living in refugee camps [22]. A Cochrane review indicated significantly lower protective efficiency of OCV in under-five children compared to children who are older than them as well as adults [23]. Vigilance for cholera cases as well as preparedness for prevention and mitigation measures for surges and mass OCV campaigns for FDMN as well as host population can reduce the threats of cholera in both the host and FDMN [24–29].

One of the limitations of the study was that these activities were DTC based as a result only those cholera cases with admissions in DTCs have been included in the study. Cholera patients with less severe disease who reported to the DTCs and received care on an out-patient basis for a brief period and those cases that occurred at the community and did not report to DTCs have not been studied. Thus, results may not be generalizable. However, the study of a fairly large number of cholera cases captured during an outbreak as well as quality laboratory performance were the strengths of the study.

Conclusion

Threats of cholera outbreaks among the FDMN are continuing due to new arrivals with compromised host susceptibility, the declining immunity to the vaccine among OCV recipients

as well as an increasing number of cohort children without any exposure to OCV. Quality surveillance and rapid microbial confirmation of provisionally diagnosed suspected cases have important public health implications in emergencies and crises. Preparedness for surges and vigilance for cholera cases should be the priority undertakings of the Health Systems of Government of Bangladesh because of existing threats of cholera in both the host and displaced populations.

Acknowledgments

We acknowledge the contribution of icddr,b’s core donors including the Government of the People’s Republic of Bangladesh, Global Affairs Canada, Canada; Swedish International Development Cooperation Agency, and UK Aid (FCDO) for their continuous support and commitment to the icddr,b’s research efforts.

Funding

This study was financially supported by UNICEF Bangladesh (donors for AWD Preparedness and Response activities include; USA (State) Bureau of Population, Refugees, and Migration (PRM), Government of Japan, Department for International Development (DFID) (now Foreign, Commonwealth & Development Office (FCDO), and Central Emergency Relief Fund for United Nations (CERF). The funding body had no role in the design of the study, data collection, analysis, and interpretation of the findings.

Declarations of Interest

None to declare.

Availability of data and material

This dataset and materials are available via the corresponding author and can be accessed on a valid request.

Author Contributions

Conceptualization: ASG Faruque, Tahmeed Ahmed, Azharul Islam Khan

Data curation: ASG Faruque, M Nasif Hossain, Soroar Hossain Khan

Formal analysis: Soroar Hossain Khan, M Nasif Hossain

Investigation: Tahmeed Ahmed, Azharul Islam Khan, ASG Faruque

Methodology: ASG Faruque, Azharul Islam Khan, SM Rafiqul Islam, Baitun Nahar, M Nasif Hossain, Soroar Hossain Khan

Project administration: SM Rafiqul Islam, Syed Asif Abdullah, Baitun Nahar, M Nasif Hossain, Soroar Hossain Khan

Supervision: M Nasif Hossain

Writing – original draft: ASG Faruque, Azharul Islam Khan, SM Rafiqul Islam, Baitun Nahar, M Nasif Hossain, Syed Asif Abdullah, Mukesh Prajapati, Minjoon Kim, Jennie Musto

Writing – review & editing: Jennie Musto, Mukesh Prajapati, Yulia Widiati, ASM Mainul Hasan, Feroz Hayat Khan, Md. Sabbir Hossain, Tahmeed Ahmed, Maya Vandenent, John David Clemens, ASG Faruque, Baitun Nahar, Azharul Islam Khan, SM Rafiqul Islam, M Nasif Hossain, Syed Asif Abdullah

References: M Nasif Hossain, Soroar Hossain Khan, ASG Faruque

References

1. Mark Bowden JC. Humanitarian Exchange Special feature Rohingya refugees in Bangladesh: the humanitarian response. 2017. Available: http://www.odihpn.org
2. Hassan M, Smith A, Walker K, Rahman M, Southworth J. Rohingya Refugee Crisis and Forest Cover Change in Teknaf, Bangladesh. Remote Sens. 2018;10: 689. doi:10.3390/rs10050689
3. Karo B, Haskew C, Khan AS, Polonsky JA, Mazhar MKA, Buddha N. World health organization early warning, alert and response system in the Rohingya crisis, Bangladesh, 2017–2018. Emerg Infect Dis. 2018;24: 2074–2076. doi:10.3201/eid2411.181237
4. Islam MM, Nuzhath T. Health risks of Rohingya refugee population in Bangladesh: A call for global attention. J Glob Health. 2018;8. doi:10.7189/jogh.08.020309
5. Kennedy J, McCoy D. WHO and the health crisis among the Rohingya people of Myanmar. Lancet. 2017;389: 802–803. doi:10.1016/S0140-6736(16)00646-2
6. Pocock NS. Imminent health crises among the Rohingya people of Myanmar The international community must act to stop “slow burning genocide.” BMJ. 2017;359: 5210. doi:10.1136/bmj.j5210
7. Villasana D. Picturing health: Rohingya refugees in Bangladesh. The Lancet. Lancet Publishing Group; 2017. pp. 2233–2242. doi:10.1016/S0140-6736(17)32862-3
8. Qadri F, Azad AK, Flora MS, Khan AI, Islam MT, Nair GB, et al. Emergency deployment of oral cholera vaccine for the Rohingya in Bangladesh. The Lancet. Lancet Publishing Group; 2018. pp. 1877–1879. doi:10.1016/S0140-6736(18)30993-0
9. Khan AI, Islam MT, Siddique SA, Ahmed S, Sheikh N, Siddik AU, et al. Post-vaccination campaign coverage evaluation of oral cholera vaccine, oral polio vaccine and measles–rubella vaccine among Forcibly Displaced Myanmar Nationals in Bangladesh. Hum Vaccines Immunother. 2019;15: 2882–2886. doi:10.1080/21645515.2019.1616502
10. Chowdhury F, Bhuiyan TR, Akter A, Bhuiyan MS, Khan AI, Hossain M, et al. Immunogenicity of a killed bivalent whole cell oral cholera vaccine in forcibly displaced

Myanmar nationals in cox's bazar, Bangladesh. PLoS Negl Trop Dis. 2020;14. doi:10.1371/journal.pntd.0007989
11. Shannon K, Hast M, Azman AS, Legros D, McKay H, Lessler J. Cholera prevention and control in refugee settings: Successes and continued challenges. PLoS Negl Trop Dis. 2019;13. doi:10.1371/journal.pntd.0007347
12. Toole MJ, Waldman RJ. The public health aspects of complex emergencies and refugee situations. Annual Review of Public Health. Annual Reviews Inc.; 1997. pp. 283–312. doi:10.1146/annurev.publhealth.18.1.283
13. WHO. Enhancing public health through mass vaccination in Cox's Bazar: two weeks of oral cholera vaccination campaign. 2019 [cited 8 Aug 2020]. Available: https://www.who.int/bangladesh/news/detail/22-12-2019-enhancing-public-health-through-mass-vaccination-in-cox-s-bazar-one-week-of-oral-cholera-vaccination-campaign
14. WHO. Cholera vaccination campaign launched to protect 635,000 people in Cox's Bazar. 2019 [cited 8 Aug 2020]. Available: https://www.who.int/bangladesh/news/detail/09-12-2019-cholera-vaccination-campaign-launched-to-protect-635-000-people-in-cox-s-bazar
15. Milton AH, Rahman M, Hussain S, Jindal C, Choudhury S, Akter S, et al. Trapped in statelessness: Rohingya refugees in Bangladesh. International Journal of Environmental Research and Public Health. MDPI AG; 2017. doi:10.3390/ijerph14080942
16. Mukul SA, Huq S, Herbohn J, Nishat A, Rahman AA, Amin R, et al. Rohingya refugees and the environment. Sills J, editor. Science (80-). 2019;364: 138–138. doi:10.1126/science.aaw9474
17. Stoll BJ, Glass RI, Huq I, Khan MU, Holt JE, Banu H. Surveillance of patients attending a diarrhoeal disease hospital in Bangladesh. Br Med J. 1982.
18. Das SK, Ahmed S, Ferdous F, Farzana FD, Chisti MJ, Latham JR, et al. Etiological diversity of diarrhoeal disease in Bangladesh. J Infect Dev Ctries. 2013;7: 900–909. doi:10.3855/jidc.3003
19. Parvin I, Shahunja KM, Khan SH, Alam T, Shahrin L, Ackhter MM, et al. Changing Susceptibility Pattern of Vibrio cholerae O1 Isolates to Commonly Used Antibiotics in the Largest Diarrheal Disease Hospital in Bangladesh during 2000–2018. Am J Trop Med Hyg. 2020;103. doi:10.4269/ajtmh.20-0058
20. Islam MT, Chowdhury F, Qadri F, Sur D, Ganguly NK. Trials of the killed oral cholera

vaccine (Shanchol) in India and Bangladesh: Lessons learned and way forward. Vaccine. Elsevier Ltd; 2020. pp. A127–A131. doi:10.1016/j.vaccine.2019.06.082

21. WHO. ENDING CHOLERA A G LO B A L R OA D M A P TO 2 0 3 0 OVERVIEW OF. 2017.

22. Azman AS, Rumunu J, Abubakar A, West H, Ciglenecki I, Helderman T, et al. Population-level effect of cholera vaccine on displaced populations, South Sudan, 2014. Emerg Infect Dis. 2016;22: 1067–1070. doi:10.3201/eid2206.151592

23. Sinclair D, Abba K, Zaman K, Qadri F, Graves PM. Oral vaccines for preventing cholera. Cochrane Database Syst Rev. 2011;2011. doi:10.1002/14651858.cd008603.pub2

24. Colombara D V., Faruque ASG, Cowgill KD, Mayer JD. Risk factors for diarrhea hospitalization in Bangladesh, 2000-2008: A case-case study of cholera and shigellosis. BMC Infect Dis. 2014;14. doi:10.1186/1471-2334-14-440

25. Leung DT, Chowdhury F, Calderwood SB, Qadri F, Ryan ET. Immune responses to cholera in children. Expert Review of Anti-Infective Therapy. NIH Public Access; 2012. pp. 435–444. doi:10.1586/eri.12.23

26. Connolly MA, Gayer M, Ryan MJ, Salama P, Spiegel P, Heymann DL. Communicable diseases in complex emergencies: Impact and challenges. Lancet. Lancet; 2004. pp. 1974–1983. doi:10.1016/S0140-6736(04)17481-3

27. Qadri F, Bhuiyan TR, Sack DA, Svennerholm AM. Immune responses and protection in children in developing countries induced by oral vaccines. Vaccine. Elsevier; 2013. pp. 452–460. doi:10.1016/j.vaccine.2012.11.012

28. Desai SN, Akalu Z, Teferi M, Manna B, Teshome S, Park JY, et al. Comparison of immune responses to a killed bivalent whole cell oral cholera vaccine between endemic and less endemic settings. Trop Med Int Heal. 2016;21: 194–201. doi:10.1111/tmi.12641

29. Levine MM. Immunogenicity and efficacy of oral vaccines in developing countries: Lessons from a live cholera vaccine. BMC Biol. 2010;8. doi:10.1186/1741-7007-8-129

Manuscript
Click here to access/download;Manuscript;Plos NTD_Coxs_Bazar_ASGF. revised (ver 2).docx

Cholera outbreak in Forcibly Displaced Myanmar National (FDMN) from a small population segment in Cox's Bazar, Bangladesh, 2019

ASG Faruque[1¶*], Azharul Islam Khan[1¶], Baitun Nahar[1], SM Rafiqul Islam[1], M Nasif Hossain[1], Syed Asif Abdullah[1], Soroar Hossain Khan[1], Md. Sabbir Hossain[2], Feroz Hayat Khan[2], Mukesh Prajapati[2], Yulia Widiati[3], ASM Mainul Hasan[3], Minjoon Kim[4], Jennie Musto[2], Maya Vandenent[4], John David Clemens[1,5], Tahmeed Ahmed[1]

[1]Nutrition and Clinical Services Division, icddr,b, Dhaka 1212, Bangladesh

[2]World Health Organization, Cox's Bazar, Bangladesh

[3]UNICEF Bangladesh, Cox's Bazar Field Office

[4]UNICEF Bangladesh Country Office

[5]UCLA Fielding School of Public Health, USA

¶ These authors Contributed equally to this work as joint first authors

Corresponding author

*ASG Faruque

Email: gfaruque@icddrb.org

Keywords. Cholera, under-five children, displaced population, emergency and crisis settings, patient management

Short Title: Cholera outbreak in displaced population

Abstract

Background: Bangladesh experienced a sudden, large influx of forcibly displaced persons from Myanmar in August 2017. A cholera outbreak occurred in the displaced population during September-December 2019. This study aims to describe the epidemiologic characteristics of cholera patients who were hospitalized in diarrhea treatment centers (DTCs) and sought care from settlements of Forcibly Displaced Myanmar Nationals (FDMN) as well as host country nationals during the cholera outbreak.

Methods*:* Diarrhea Treatment Center (DTC) based surveillance was carried out among the FDMN and host population in Teknaf and Leda DTCs hospitalized for cholera during September-December 2019.

Results: During the study period, 147 individuals with cholera were hospitalized. The majority, 72% of patients reported to Leda DTC. Nearly 65% sought care from FDMN settlements. About 47% of the cholera individuals were children less than 5 years old and 42% were aged 15 years and more. Half of the cholera patients were females. FDMN often reported from Camp # 26 (45%), followed by Camp # 24 (36%), and Camp # 27 (12%). Eighty-two percent of the cholera patients reported watery diarrhea. Some or severe dehydration was observed in 65% of cholera individuals. Eighty-one percent of people with cholera received pre-packaged ORS at home. About 88% of FDMN cholera patients reported consumption of public tap water. Pit latrine without water seal was often used by FDMN cholera individuals (78%).

Conclusion: Vigilance for cholera patients by routine surveillance, preparedness, and response readiness for surges and oral cholera vaccination campaigns can alleviate the threats of

cholera.

Author Summary

Bangladesh observed a large-scale arrival of forcibly displaced individuals from Myanmar in August 2017. The Bangladesh Government, UN agencies, and international and national non-governmental organizations responded immediately with extensive humanitarian response. However, threats of cholera outbreaks were prevailing. The Government of Bangladesh as lead, with technical support from icddr,b collaborating with international agencies undertook a massive oral cholera vaccination (OCV) campaign immediately as a pre-emptive measure to alleviate threats of the cholera outbreak. Despite that mass OCV campaign, threats of cholera outbreak were existing due to new arrivals of the displaced population with compromised host susceptibility, frequent visits to settlements by Bangladesh nationals without exposure to OCV, and the declining vaccine immunity among OCV recipients as well as an increasing number of cohort children without any exposure to OCV. The population faced a cholera outbreak during September-December 2019. This study aims to describe the characteristics of cholera patients, their care-seeking pattern, camp-wise distribution, source of drinking water, sanitation facility, OCV status, and share the experiences from effective interventions to prevent a cholera outbreak. Vigilance for cholera patients by routine surveillance, preparedness for both preventive and control measures, and response readiness for surges and OCV campaigns can alleviate the threats of cholera.

Introduction

In August 2017, Bangladesh witnessed a sudden influx of an estimated 745,000 Forcibly Displaced Myanmar Nationals (FDMN) including more than 400,000 children within 17 weeks from neighboring Rakhine state in Myanmar who settled in the Cox's Bazar district situated in the south-east of the country. Their journey to Bangladesh was hazardous with limited access to food and water, often had diverse injuries and illnesses, crossed through jungles and mountainous terrain and finally, most of them had a boat ride to cross the Naf river while some confronted risky sea voyage across the Bay of Bengal. The Bangladesh Government, UN agencies, and a large number of international and national non-governmental organizations (NGOs) reacted immediately with a large-scale humanitarian response. Camps were established quickly but soon humanitarian agencies started struggling to meet the exorbitant demand for assistance and supplies. The displaced population urgently needed critical supplies like medicine, clean water, food, and shelter with special attention to children, women, the elderly, and disabled individuals. Many of the hurriedly built camps were vulnerable to monsoon flooding and storm surges. Those families who started living in hillsides were prone to landslides. Latrines and shallow and deep tube wells were constructed to protect against public health issues and ensure access to clean water. However, because of the arrival of a large number of displaced populations and the presence of insufficient lifesaving infrastructures of sanitation, like latrines and waterpoints, the environment soon became a breeding place for waterborne diseases including acute watery diarrhea, cholera, and shigellosis. These risks were further heightened by high population density in camps and an excess number of severely malnourished children who often yield more quickly to preventable and treatable diseases as well as outbreaks of acute watery diarrhea (AWD), cholera, and shigellosis [1–7].

Almost immediately, following the huge influx and settlement of these displaced populations,

UNICEF-Bangladesh and icddr,b jointly conducted a brief field assessment in the Ukhia and Teknaf sub-districts of Cox's Bazar. The assessment anticipated potential threats of diarrheal disease outbreaks including cholera and shigellosis, and strategies were immediately set to initiate mitigation measures. A partnership between icddr,b, and UNICEF under the umbrella of Health Sector targeted (i) training doctors, nurses, and community health workers of the government and NGO run facilities serving FDMN in the settlements as well as host population living in the neighborhood housing; (ii) managing people with dehydrating diarrheal episodes and associated malnutrition through a network of five diarrhea treatment centers (DTCs); and carrying out DTC based diarrheal disease surveillance as it is known to be critical for early detection of outbreaks. Activities of the diarrheal disease surveillance team included data collection, a one-step rapid diagnostic test for the presence of *Vibrio cholerae* in stool specimen of hospitalized patients, and microbial tests to detect common enteric pathogens including *Vibrio cholerae* by submitting fecal specimens directly as well as after inoculation into Cary-Blair Transport Medium to the Clinical Microbiology Laboratory of icddr,b in Dhaka, Bangladesh.

The Government of Bangladesh as lead, with technical support from icddr,b collaborating with international agencies, and international and national NGOs under the wider platform of Health Sector, undertook a massive oral cholera vaccination (OCV) campaign immediately as a pre-emptive measure to alleviate threats of cholera outbreaks [8–10]. Despite that mass OCV campaign, threats of cholera outbreaks among FDMN were existing due to new arrivals of the displaced population with compromised host susceptibility, frequent visits to settlements by Bangladesh nationals living in the neighboring community without exposure to OCV, and the decay of vaccine immunity in OCV recipients as well as an increasing number of cohort children

without any exposure to OCV. Preparedness for preventive and control measures to combat surges and vigilance for people with cholera was the most important public health priorities because of prevailing threats of cholera in both the host and displaced population [11,12].

icddr,b, and UNICEF jointly organized a dissemination session for the local stakeholders on their activities for the FDMN living in the settlements in March 2019. Between September and December 2019, there have been 147 people with culture-confirmed cholera who presented and subsequently hospitalized with acute dehydrating diarrhea episodes in Leda and Teknaf DTCs. Thus, it became essential to share this cholera outbreak control experience with policymakers, public health teams, program managers, academia, and wider stakeholders acquired from a strategy in an emergency and crisis setting. Such experience sharing is not a common and widespread phenomenon, particularly in humanitarian emergencies. An update of this kind is likely to enable stakeholders to undertake necessary preparedness to prevent cholera outbreaks from occurring and to respond successfully when the outbreaks have occurred.

In late September 2019, two cholera patients for the first time after two years of the arrival of FDMN were detected in Teknaf DTC which is run by icddr,b. They sought care from settlements (one from Camp # 25 and the other from Camp # 26). Such an incident was reported immediately to the Epidemiology Team Lead and Early Warning, Alert and Response System (EWARS) of WHO-Cox’s Bazar, as well as UNICEF-Cox’s Bazar. The next day, Cox’s Bazar Health Sector’s Joint Assessment Team (JAT) consisting of Health and WASH Sector partners investigated the hotspots and affected camps. The JAT reported worsening hygiene practices and sanitary conditions as a result of an acute shortage of safe drinking water, and the use of stagnant

contaminated water for domestic purposes. Several recommendations were made on that day including hygiene promotion in the hot spots, desludging of latrines as soon as possible, distribution of water purifying tablets, pre-packaged ORS, soap, and chlorine by the WASH Sector, and availability of a handwashing facility in the latrine areas. The stagnant contaminated pools of water were fenced to prevent access to it by people living in its surroundings. Urgent refresher training on risk assessment for health teams was recommended. Within 24 hours, one temporarily closed down DTC in Leda nearby by the affected settlements was reopened to serve the increasing number of AWD patients.

The Health Systems of Bangladesh Government continued collaboration with WHO-Cox's Bazar in streamlining activities of EWARS, actively involved in strengthened monitoring of the individuals with AWD and cholera in the camps for early detection and response to outbreaks. Institution of immediate alleviation measures included the supply of safe drinking water and improvement of the sanitation system. To ensure adequate clinical management of AWD individuals following a standard management protocol, the existing network of DTCs was strengthened by UNICEF-Cox's Bazar. WHO and the Health Sector recommended that those patients presenting to the out-patient clinics with dehydrating diarrhea should be immediately referred to Diarrhea Treatment Centres (DTCs) run by icddr,b, or, if there were no DTCs nearby, to primary health care centers (PHCs) with isolation facilities. Leda DTC (14 beds) and Teknaf DTC (30 beds) located in the neighborhood of settlements remained open as usual round-the-clock. Six batches of the health workforce were immediately trained by icddr,b on the clinical management of AWD individuals. Community health workers were also assigned by UNICEF-Cox's Bazar in outreach activities including promotion of good hygiene practices and combatting

diarrhea episodes at the household level with the use of pre-packaged ORS as soon as there is the onset of these episodes [13–16].

Preparations and response readiness were undertaken for the acceleration of the existing cholera vaccination campaign as an increasing trend of dehydrating diarrhea patients in DTCs was revealed. As a result, the International Coordinating Group for Cholera Vaccine (ICG) Secretariat approved a request for additional 1.2 million doses of OCV. Ministry of Health and Family Welfare, Bangladesh playing the leading role with the support of WHO, UNICEF, and other partners, the campaign started vaccinating those individuals living in the neighborhood host community but yet to receive any OCV. The OCV campaign (including operational cost) was funded by GAVI, the Vaccine Alliance. The vaccination operation aimed mostly to reach displaced children aged 12-59 months. In the host community, the campaign looked for any person aged 1 year or more, because approximately 80% of host community people residing near the settlements were never targeted to receive OCV in previous campaigns although they were equally vulnerable like the FDMN [13,14].

This paper aims to (i) describe the characteristics of cholera patients including that of FDMN care seekers, their reporting pattern to DTCs, camp-wise distribution, and OCV status, (ii) compare drinking water sources and toilet use pattern between FDMN and host community cholera individuals, (iii) describe comparative clinical and demographic characteristics between cholera individuals who sought care from Cox's Bazar DTCs, and Dhaka Hospital of icddr,b during the same period, and (iv) share the experiences that were obtained from this cholera outbreak that occurred in a small segment of the FDMN living in settlements of Cox's Bazar, Bangladesh.

Methods

Ethics statement

The data collection process of this study was part of the ongoing activities entitled: *Surveillance for etiologic agents, care-seeking behavior, the status of IYCF and WASH practices among patients or their caregivers from Rohingya refugees as well as host population in Cox's Bazar district attending icddr,b operated Diarrhea Treatment Centers* was approved by icddr,b's (International Centre for Diarrhoeal Disease Research, Bangladesh) IRB (PR-17111; December 5, 2017) comprising Research Review Committee (RRC) and Ethical Review Committee (ERC). Voluntary written informed consent was obtained from the parent/guardian before starting the interviewing process.

Setting and study population

This was a DTC-based cross-sectional diarrheal disease surveillance for FDMN and host community individuals hospitalized in DTCs located in Leda and Teknaf from September to December 2019.

Stool sample collection, rapid diagnostic testing, and laboratory methods

Routine enteric pathogen detection activities that included a collection of a single stool specimen (of at least 3 g) directly from the patients following hospitalization were ongoing in DTCs. Soon after collection, a one-step rapid diagnostic test was performed by SD BIOLINE cholera antigen

O1/O139 (44FK30) test kit, supplied by WHO-Cox's Bazar, which is an immunochromatographic test for the qualitative detection of *Vibrio cholerae* O1/O139 in human stool specimens (manufactured by STANDARD DIAGNOSTICS, INC located in Suwon city, Kyonggi province, Republic of Korea). To facilitate microbial culture to confirm the rapid diagnostic test results; the provisionally diagnosed specimens (the stool) of cholera patients were inoculated into Cary-Blair Transport Medium; and the medium was then sent as soon as possible to the Clinical Microbiology Laboratory, icddr,b, based in Dhaka, Bangladesh to isolate the colony as well as perform antibiotic susceptibility tests with immediate sharing of the results to the concerned DTC, Epidemiology Team Lead of WHO-Cox's Bazar and UNICEF-Cox's Bazar. Other non-positive by rapid diagnostic test specimens were submitted routinely once or twice a week [17–19].

Data collection

In daily monitoring, evaluation, and reporting, the present study followed DTC based diarrheal disease surveillance system (DDSS) in Teknaf and Leda for culture confirmed cholera patients during September-December 2019. Ongoing data collection by trained research assistants entailed administering structured questionnaires, from all hospitalized patients in DTCs and/or their attendants to gather information such as presenting clinical features, socioeconomic and demographic contexts, water, sanitation and hygiene, housing and its surrounding environment, feeding practices, particularly of 0-35 months old, and use of drugs and pre-packaged ORS at home before coming to DTCs that continued serving round-the-clock. During the interview of host population, research assistants were comfortable with the native Bengali language; however, when needed particularly in case of FDMN they received assistance of DTC staff members who

understood the dialect of FDMN, familiar with their culture, day to day living patterns and housing environments in settlements.

Statistical analysis

Data were analyzed by STATA (StataCorp version 13) and analyses included descriptive methods. Variables were described using frequencies with percentages. Exposure categories were compared using the Chi Square test for categorical variables. Relevant data from the ongoing DDSS database of Dhaka Hospital were extracted for the period September-December, 2019 for a comparative analysis of clinical and demographic profiles of visiting culture-proven cholera cpatients between Cox's Bazar DTCs and Dhaka Hospital of icddr,b.

Results

Between September and December 2019, there were147 culture-confirmed cholera patients presented and were subsequently hospitalized with acute dehydrating diarrhea episodes in Leda and Teknaf DTCs. The majority, 72% of cholera individuals reported to Leda DTC. Nearly 65% of these cholera patients sought care from FDMN settlements. FDMN often reported to DTCs from Camp # 26 (45%), followed by Camp # 24 (36%), and Camp # 27 (12%). About 94% of the cholera patients from the host community and 65% of the cholera individuals from FDMN living in settlements did not receive any OCV before their onset of culture-proven cholera episodes (Table 1). Overall, these DTCs served during the outbreak an estimated 22% of both FDMN living in settlements and host country nationals residing in the neighborhood (Figure 1).

Figure 1. Distribution of and camps from where cholera patients reported to Leda and Teknaf DTCS, September-December 2019, Teknaf, Cox's Bazar, Bangladesh

Table 1 Distribution of characteristics of culture-confirmed cholera patients (n=147) in Leda and Teknaf DTCs in Cox's Bazar settlements, September-December 2019

Variables name	n (%)
**Sought care from**	
Leda DTC	106 (72.1)
Teknaf	41 (27.9)
**Currently living in**	
Settlements	95 (64.6)
Host community	52 (35.4)
**From settlements**	
Camp # 26	43 (45.3)
Camp # 24	34 (35.8)
Camp # 27	11 (11.6)
Camp # 25	4 (4.2)
Camp # 15	2 (2.1)
Camp # 23	1 (1.1)
**Not exposed to OCV**	
FDMN	62 (65.3)
Host community individuals	49 (94.2)

DTC: Diarrhea treatment center; OCV: Oral cholera vaccine

The major sources of drinking water of the hospitalized displaced cholera individuals were public tap installed in the settlements, deep tube-well, and shallow tube well. Use of public tap water was significantly more frequent in cholera patients from settlements than from the host community (88% vs. 10%; $p<0.001$). However, the use of deep tube well (6% vs. 21%; $p=0.005$) and shallow tube well (2% vs. 54%; $p<0.001$) water was significantly less common in the cholera patients from settlements. Nearly 78% of the displaced cholera patients used pit latrines without water seal as opposed to 44% of the individuals with cholera from the host community ($p<0.001$). However, the use of a pit latrine with a water seal was identical in both groups (Table 2).

Table 2 Water source and toilet use by the culture-confirmed cholera patients in Leda and Teknaf DTCs in Cox's Bazar settlements, September-December 2019

Variables	FDMN *n=95* (%)	Host community *n=52* (%)	P-value
**Water source**			
Public tap	84 (88.4)	5 (9.6)	<0.001
Deep tube well	6 (6.3)	11 (21.2)	0.005
Shallow tube well	2 (2.1)	28 (53.8)	<0.001
Others	3 (3.2)	8 (15.4)	0.005
**Toilet use pattern**			
Pit latrine without water seal	74 (77.9)	23 (44.2)	<0.001
Pit latrine with water seal	21 (22.1)	12 (23.1)	0.819
Others	0 (0.0)	17 (32.7)	<0.001

During September-December 2019, a total of 216 culture-confirmed cholera individuals were hospitalized in icddr,b's Dhaka Hospital, and none had received OCV. During the same period, DTC logs reported the admission of 147 culture-proven cholera patients in Leda and Teknaf DTCs. Among these cholera patients, infants ($p<0.001$) and overall children <5 years old ($p<0.001$) presented more frequently to the DTCs (functioning to treat FDMN living in settlements as well as host community individuals) compared to cholera children presenting to Dhaka Hospital from Dhaka city and its suburbs (47% vs. 12%; $p<0.001$). However, for individuals aged 15 years and higher, more cholera patients reported to Dhaka Hospital as opposed to cholera patients living in settlements and seeking care from DTCs (76% vs. 42%; $p<0.001$) (Table 3). Significantly more female cholera patients visited DTCs as opposed to female cholera patients presenting to Dhaka Hospital (50% vs. 38%, $p<0.043$). People with cholera in Dhaka Hospital more commonly presented with watery diarrhea than cholera patients of DTCs (100% vs. 82%, $p<0.001$), sought care more frequently with some or severe dehydration (98% vs. 65%, $p<0.001$), and had more access to ORS at home before seeking care (91% vs. 81%, $p<0.010$) (Table 3).

Table 3 Age stratified cholera patients in Dhaka Hospital and DTCs in Cox's Bazar settlements, September-December 2019

Variables	Dhaka hospital	DTCs in settlements	p-value

	n=216 (%)	n=147 (%)	
**Age (Year)**			
<1	3 (1.4)	14 (9.5)	<0.001
<5	25 (11.6)	69 (46.9)	<0.001
5-14	28 (13.0)	17 (11.6)	0.814
15 and more	163 (75.5)	61 (41.5)	<0.001
**Range**	7 months – 74 years	3 months – 85 years	
**Female**	83 (38.4)	73 (49.7)	0.043
**Duration of diarrhea**			
<1 day	153 (70.8)	100 (68.0)	0.300
1-3 days	57 (26.4)	38 (25.9)	0.994
4 days and more	6 (2.8)	9 (6.1)	0.192
**Watery stool**	216 (100.0)	120 (81.6)	<0.001
**Some or severe dehydration**	211 (97.7)	95 (64.6)	<0.001
**Pre-packaged ORS us at home**	196 (90.7)	119 (81.0)	0.010

DTC: Diarrhea treatment center; ORS: Oral rehydration solution

Discussion

Humanitarian emergencies increase the risk of infectious disease transmission including cholera and shigellosis, and the prevalence of other health conditions such as severe undernutrition. In a given similar scenario with preparedness for both preventive and control measures and response readiness, our observations highlighted the vital role of an effective disease surveillance system that continually generates essential epidemiologic data for effective strategy formulation. Such a system is critical for early detection of disease outbreaks before any spread to other family members as well as individuals living in the neighborhood, unnecessarily costing lives and challenging the disease control efforts. Thus, our ongoing DTC-based diarrheal disease surveillance system with timely laboratory back-up and immediate reporting to all concerned agencies was noteworthy in this emergency and crisis setting. The surveillance system was involved not only in collecting reliable data since the inception of the DTC network but also in reporting immediately to help significantly in anticipating and detecting early potential cholera outbreaks. Findings from surveillance system guided intervention strategies that lead to the timely undertaking of preventive measures and the preparedness that included training of health care staff,

opening of temporarily closed down DTC, strengthening of existing DTCs, outreach activities, and prepositioning of supplies as well as additional human resources. Other additional vital strategies undertaken were inter-sectoral collaboration, strengthening of preventive and control measures (regular monitoring of the quality of drinking water sources at waterpoints and household level, sanitation as well hygiene) as well as OCV campaigns. Efforts further emphasized preparedness for surges and vigilance for cholera patients which was the priority undertakings of the Health Systems of Government of Bangladesh because of existing threats of cholera in both the host and displaced populations in emergency and current settings.

Additionally, surveillance data helped in identifying vulnerable populations living in high-risk areas who might have been benefitted from preventive OCV use. Thus, reliable epidemiological data was critical in the efficient implementation of preventive as well as control measures.

The present study observed that 94% of the host community individuals and two-third of the FDMN with laboratory-confirmed cholera were not exposed to OCV before getting hospitalized with AWD. A recent experience from Bangladesh and India indicated that the protective efficacy of Shanchol OCV (produced in India) among those more than five years against cholera is 53-65%. The study mentioned the positive role of OCV as a pre-emptive measure in endemic settings, in natural or man-made disasters even in disruptive situations with a breakdown of WASH and absence of other disease control and public health measures [20]. WHO and Global Task Force for Cholera Control (GTFCC) recommend that a comprehensive multi-sectoral involvement is important for the successful elimination of cholera [21]. Mass OCV campaigns with high coverage are feasible even after the arrival of a large number of displaced populations in a distressed state in resource poor settings like Bangladesh [8,9]. According to another study, OCV induced optimal

immune responses in FDMN adults and children which were similar to that observed in Bangladesh's population of diverse age groups or individuals living in other cholera endemic countries [10]. In Sudan among the displaced populations, the risks for cholera were considerably higher among children less than five years living in refugee camps [22]. A Cochrane review indicated significantly lower protective efficiency of OCV in under-five children compared to children who are older than them as well as adults [23]. Vigilance for cholera individuals as well as preparedness for prevention and mitigation measures for surges and mass OCV campaigns for FDMN as well as host population can reduce the threats of cholera in both the host and FDMN [24–29].

In this study, we have explored the clinical, demographic, and hygienic practices of the displaced as well as the host population living in settlements and neighboring host communities. The findings of this study have public health implications and may be useful for the Health System of the Government of Bangladesh for anticipation, preparedness, and implementation of preventive and mitigation measures in settings with public health threats such as endemic disease surges like cholera or it is breaking out into epidemic proportions. Additionally, vigilance for cause-specific diarrhea surges in both the populations such as host and FDMN is critical. Several findings related to care-seeking from DTCs were noteworthy. Unlike Dhaka hospital, children living in settlements and host communities were more often hospitalized for culture-proven cholera episodes than their peers from Dhaka city and its suburbs. These observations underscore the need for OCV campaigns. Females aged 15 years and higher living in settlements were more often hospitalized with cholera than their peers seeking care from Dhaka Hospital. This may be due to the increased vulnerability of females living in settlements to cholera because of their higher compromised

immunity or excess exposure to contaminated water and food during household activities. Excess reporting of male cholera patients in Dhaka Hospital may be due to increased mobility of male individuals as well as their frequent exposures to day-time unhygienic outdoor street-side meals or snacks from vendors in the overcrowded megacity.

ORS use at home was significantly lower in the cholera patients seeking care from DTCs than those cholera individuals living in Dhaka city and its suburbs. A big factor limiting people's use of ORS is their knowledge of when and how to use this vital tool. Major limitations of outreach activities in this scenario may include less promotion and access to ORS packets at the household or community level in settlements, because of less organized outreach activities. Additionally, lack of appropriate health education measures to make FDMN knowledgeable about ORS use particularly when to start, how to prepare, how much to be taken, and how long to be continued. All these more effective attempts may motivate FDMN to enhance their appropriate use of ORS at the household level before coming to DTCs.

Access to more safe water (chlorinated water supplied through taps installed) was observed in settlements mostly for FDMN as provided by international agencies and NGOs. However, their access to deep and shallow tube well water was less commonly observed compared to that of admissions from the host community. It is important that treatment of water is a vital tool for providing safe water when tube wells are inadequate in meeting the needs of the displaced population in emergency and crisis settings.

Cholera patients with significantly more frequent watery stool and with more common evidence of some or severe dehydration in Dhaka hospital could be due to more full-blown clinical features of cholera episodes which may be because of larger inoculum size that may be ingested by those living in the more contaminated environment particularly in slums with gross lack of water and sanitation services as well as worsening hygienic practices in Dhaka city and its suburbs.

icddr,b followed its expertise gathered from its hospital-based Diarrheal Disease Surveillance System (DDSS) which is in operation in icddr,b's urban Dhaka (since 1979), and rural Matlab (since 1999) facilities. The Diarrheal Disease Surveillance System (DDSS) at Dhaka Hospital enrolls a 2% systematic sample of patients reporting to the triage area. Patients seeking care from the Matlab Hospital who are residents of the Health Demographic Surveillance System (HDSS) area are enrolled into the DDSS. Trained enumerators using structured questionnaires interview patients and/or their attendants to collect relevant information. Microbiological assessments are performed to identify common diarrheal pathogens and document the microbial susceptibility pattern of the bacterial pathogens. The activity offers useful information to hospital clinicians in their clinical decision-making courses and empowers icddr,b to detect the emergence of new enteric pathogens and early recognition of outbreaks and their locations, thereby guiding the host government to take suitable preventive and control measures [17–19].

There was an absence of comparable diarrhea treatment facilities in the settlements which not only providing quality care but also examining stool specimens for diarrheagenic organisms following standard laboratory methods. We needed data for comparison of presenting clinical and demographic features of hospitalized cholera patients (such as age sex, duration of diarrhea, watery stool, dehydration status, and pre-packaged ORS use) from Leda and Teknaf DTCs with that of a facility that has a track record of diarrheal disease surveillance system and treating hospitalized

cholera patients who are seeking care from such a facility that does not charge for the services, provides quality care mostly to those attending from poor socio-economic contexts, remains open round-the-clock, and can efficiently handle sudden upsurges of patients including individuals with cholera presenting often in a dehydrated state in a relatively large number and the facility has a back-up laboratory for routine fecal specimen examinations following standard methods for detection and characterization of causative enteric organisms including *V. cholerae.*

This study has few limitations and one of the limitations was these activities were DTC based as a result only those cholera individuals with admissions in DTCs have been included in the study. Cholera patients with less severe disease who reported to the DTCs and received care on an outpatient basis for a brief period and those patients who developed cholera at the community and did not report to DTCs have not been studied. Thus, results may not be generalizable. However, the study of a fairly large number of cholera patients captured during an outbreak as well as quality laboratory performance were the strengths of the study.

Conclusion

Threats of cholera outbreaks among the FDMN are continuing due to new arrivals with compromised host susceptibility, the declining immunity to the vaccine among OCV recipients as well as an increasing number of cohort children without any exposure to OCV. Quality surveillance and rapid microbial confirmation of provisionally diagnosed suspected individuals with cholera have important public health implications in emergencies and crises. Continued preventive and control measures, preparedness and response readiness for surges, and vigilance for cholera patients should be the priority undertakings of the Health Systems of Government of Bangladesh because of existing threats of cholera in both the host and displaced populations.

Acknowledgments

We acknowledge the contribution of icddr,b's core donors including the Government of the People's Republic of Bangladesh, Global Affairs Canada, Canada; Swedish International Development Cooperation Agency, and UK Aid (FCDO) for their continuous support and commitment to the icddr,b's research efforts.

References

1. Mark Bowden JC. Humanitarian Exchange Special feature Rohingya refugees in Bangladesh: the humanitarian response. 2017. Available: http://www.odihpn.org
2. Hassan M, Smith A, Walker K, Rahman M, Southworth J. Rohingya Refugee Crisis and Forest Cover Change in Teknaf, Bangladesh. Remote Sens. 2018;10: 689. doi:10.3390/rs10050689
3. Karo B, Haskew C, Khan AS, Polonsky JA, Mazhar MKA, Buddha N. World health organization early warning, alert and response system in the Rohingya crisis, Bangladesh, 2017–2018. Emerg Infect Dis. 2018;24: 2074–2076. doi:10.3201/eid2411.181237
4. Islam MM, Nuzhath T. Health risks of Rohingya refugee population in Bangladesh: A call for global attention. J Glob Health. 2018;8. doi:10.7189/jogh.08.020309
5. Kennedy J, McCoy D. WHO and the health crisis among the Rohingya people of Myanmar. Lancet. 2017;389: 802–803. doi:10.1016/S0140-6736(16)00646-2
6. Pocock NS. Imminent health crises among the Rohingya people of Myanmar The

international community must act to stop “slow burning genocide.” BMJ. 2017;359: 5210. doi:10.1136/bmj.j5210

7. Villasana D. Picturing health: Rohingya refugees in Bangladesh. The Lancet. Lancet Publishing Group; 2017. pp. 2233–2242. doi:10.1016/S0140-6736(17)32862-3

8. Qadri F, Azad AK, Flora MS, Khan AI, Islam MT, Nair GB, et al. Emergency deployment of oral cholera vaccine for the Rohingya in Bangladesh. The Lancet. Lancet Publishing Group; 2018. pp. 1877–1879. doi:10.1016/S0140-6736(18)30993-0

9. Khan AI, Islam MT, Siddique SA, Ahmed S, Sheikh N, Siddik AU, et al. Post-vaccination campaign coverage evaluation of oral cholera vaccine, oral polio vaccine and measles–rubella vaccine among Forcibly Displaced Myanmar Nationals in Bangladesh. Hum Vaccines Immunother. 2019;15: 2882–2886. doi:10.1080/21645515.2019.1616502

10. Chowdhury F, Bhuiyan TR, Akter A, Bhuiyan MS, Khan AI, Hossain M, et al. Immunogenicity of a killed bivalent whole cell oral cholera vaccine in forcibly displaced Myanmar nationals in cox’s bazar, Bangladesh. PLoS Negl Trop Dis. 2020;14. doi:10.1371/journal.pntd.0007989

11. Shannon K, Hast M, Azman AS, Legros D, McKay H, Lessler J. Cholera prevention and control in refugee settings: Successes and continued challenges. PLoS Negl Trop Dis. 2019;13. doi:10.1371/journal.pntd.0007347

12. Toole MJ, Waldman RJ. The public health aspects of complex emergencies and refugee situations. Annual Review of Public Health. Annual Reviews Inc.; 1997. pp. 283–312. doi:10.1146/annurev.publhealth.18.1.283

13. WHO. Enhancing public health through mass vaccination in Cox’s Bazar: two weeks of oral cholera vaccination campaign. 2019 [cited 8 Aug 2020]. Available:

https://www.who.int/bangladesh/news/detail/22-12-2019-enhancing-public-health-through-mass-vaccination-in-cox-s-bazar-one-week-of-oral-cholera-vaccination-campaign

14. WHO. Cholera vaccination campaign launched to protect 635,000 people in Cox's Bazar. 2019 [cited 8 Aug 2020]. Available: https://www.who.int/bangladesh/news/detail/09-12-2019-cholera-vaccination-campaign-launched-to-protect-635-000-people-in-cox-s-bazar

15. Milton AH, Rahman M, Hussain S, Jindal C, Choudhury S, Akter S, et al. Trapped in statelessness: Rohingya refugees in Bangladesh. International Journal of Environmental Research and Public Health. MDPI AG; 2017. doi:10.3390/ijerph14080942

16. Mukul SA, Huq S, Herbohn J, Nishat A, Rahman AA, Amin R, et al. Rohingya refugees and the environment. Sills J, editor. Science (80-). 2019;364: 138–138. doi:10.1126/science.aaw9474

17. Stoll BJ, Glass RI, Huq I, Khan MU, Holt JE, Banu H. Surveillance of patients attending a diarrhoeal disease hospital in Bangladesh. Br Med J. 1982.

18. Das SK, Ahmed S, Ferdous F, Farzana FD, Chisti MJ, Latham JR, et al. Etiological diversity of diarrhoeal disease in Bangladesh. J Infect Dev Ctries. 2013;7: 900–909. doi:10.3855/jidc.3003

19. Parvin I, Shahunja KM, Khan SH, Alam T, Shahrin L, Ackhter MM, et al. Changing Susceptibility Pattern of Vibrio cholerae O1 Isolates to Commonly Used Antibiotics in the Largest Diarrheal Disease Hospital in Bangladesh during 2000–2018. Am J Trop Med Hyg. 2020;103. doi:10.4269/ajtmh.20-0058

20. Islam MT, Chowdhury F, Qadri F, Sur D, Ganguly NK. Trials of the killed oral cholera vaccine (Shanchol) in India and Bangladesh: Lessons learned and way forward. Vaccine. Elsevier Ltd; 2020. pp. A127–A131. doi:10.1016/j.vaccine.2019.06.082

21. WHO. ENDING CHOLERA A G LO B A L R OA D M A P TO 2 0 3 0 OVERVIEW OF. 2017.

22. Azman AS, Rumunu J, Abubakar A, West H, Ciglenecki I, Helderman T, et al. Population-level effect of cholera vaccine on displaced populations, South Sudan, 2014. Emerg Infect Dis. 2016;22: 1067–1070. doi:10.3201/eid2206.151592

23. Sinclair D, Abba K, Zaman K, Qadri F, Graves PM. Oral vaccines for preventing cholera. Cochrane Database Syst Rev. 2011;2011. doi:10.1002/14651858.cd008603.pub2

24. Colombara D V., Faruque ASG, Cowgill KD, Mayer JD. Risk factors for diarrhea hospitalization in Bangladesh, 2000-2008: A case-case study of cholera and shigellosis. BMC Infect Dis. 2014;14. doi:10.1186/1471-2334-14-440

25. Leung DT, Chowdhury F, Calderwood SB, Qadri F, Ryan ET. Immune responses to cholera in children. Expert Review of Anti-Infective Therapy. NIH Public Access; 2012. pp. 435–444. doi:10.1586/eri.12.23

26. Connolly MA, Gayer M, Ryan MJ, Salama P, Spiegel P, Heymann DL. Communicable diseases in complex emergencies: Impact and challenges. Lancet. Lancet; 2004. pp. 1974–1983. doi:10.1016/S0140-6736(04)17481-3

27. Qadri F, Bhuiyan TR, Sack DA, Svennerholm AM. Immune responses and protection in children in developing countries induced by oral vaccines. Vaccine. Elsevier; 2013. pp. 452–460. doi:10.1016/j.vaccine.2012.11.012

28. Desai SN, Akalu Z, Teferi M, Manna B, Teshome S, Park JY, et al. Comparison of immune responses to a killed bivalent whole cell oral cholera vaccine between endemic and less endemic settings. Trop Med Int Heal. 2016;21: 194–201. doi:10.1111/tmi.12641

29. Levine MM. Immunogenicity and efficacy of oral vaccines in developing countries:

Lessons from a live cholera vaccine. BMC Biol. 2010;8. doi:10.1186/1741-7007-8-129

Figure

Click here to access/download;Figure;Fig 1 With DTC.tif

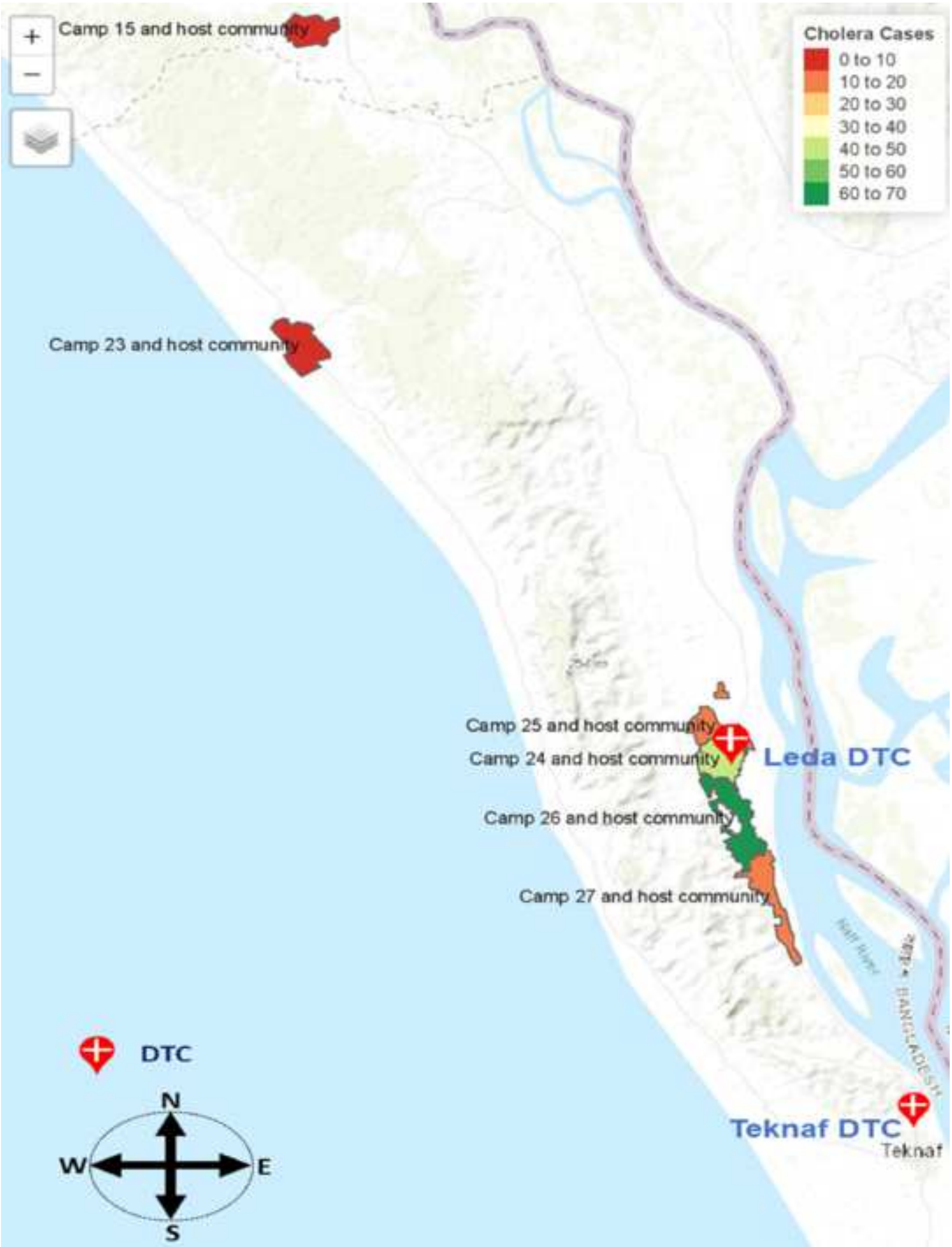

Click here to access/download
Supporting Information - Compressed/ZIP File Archive
Fig 1.zip

Point-by-point responses to reviewer's comment:

Methods

Reviewer #1: Overall, the objectives were clearly stated in the background section of the manuscript. However, I found the objectives, as written in the abstract, to be too long and comprehensive to follow. I would suggest shortening the objectives to a simple statement about collecting cholera epidemiology in a sample of refugees and host country nationals.

Response: Thank you for your kind suggestions. The objectives in the abstract have been shortened to a simple statement. Line 27-30.

Comment: The study design was appropriate for these objectives. I found the methods section to be a little confusing. The first half (lines 139-185) of the methods appeared to be more of a background about the outbreak than the specific methods of the study. I strongly suggest that portions of that section be moved to the background, with others (how did they respond to the outbreak) moved to the conclusion. That way the results are framed around those two pieces of context.

Response: Many thanks for your valuable suggestions. Suggested portions of the Methods section have been moved to the Introduction section. As also suggested, other portions have been moved to the Discussion section. Line 128-160.

Comment: Further, this first half of the methods had a sub-header (setting and study population), but the rest of the methods section did not. I suggest including additional headers to help guide the reader and follow what to me was a very complex section of the paper. These could include descriptions of the surveillance systems, survey and lab methods, statistical methods, and ethical review.

Response: Our heartfelt thanks for the valuable comments and suggestions. We have followed your suggestions and included other sub-headings like (i) Stool sample collection, rapid diagnostic testing, and laboratory methods, (ii) Data collection, and (iii) Statistical Analysis. Line 194-235

Comment: I have some specific comments/questions:
-Why was Dhaka used as a comparison sample rather than a nearby clinic? One would expect patients in the high-density urban center of Bangladesh to be very different from refugees living in camps. I applaud the inclusion of host nationals living in nearby settlements, but do not understand the inclusion of this comparator group.

Response: Thank you once again for your thoughtful concerns. Written below are our explanations for using Dhaka as a comparison sample. We are delighted to have your appreciation for the inclusion of host community in our analysis.

There was an absence of comparable diarrhea treatment facilities in the settlements which not only providing quality care but also routinely examining stool specimens for diarrheagenic enteric

organisms following standard laboratory methods. We needed data for comparison of presenting clinical and demographic features of hospitalized cholera patients (such as age, sex, duration of diarrhea, watery stool, dehydration status, and pre-packaged ORS use at home before reporting) from Leda and Teknaf DTCs with that of a facility that has a track record of diarrheal disease surveillance system and treating hospitalized cholera patients who are seeking care from such a facility that does not charge for providing quality care mostly from those attending from poor socio-economic contexts, remains open round-the-clock, and can efficiently handle sudden upsurges of patients including individuals with cholera presenting often in a dehydrated state in relatively large number and the facility has back-up laboratory support for routine fecal specimen examinations following standard methods for detection and characterization of causative enteric organisms including *V. cholerae.* Line 386-397.

Comment: Some additional context on the number of camps, number of DTCs serving those camps, and how far they are from Dhaka would be helpful. As someone with limited knowledge of Bangladesh, this context would help me understand the context of the results.

Response: Thank you. There were 34 camps, 8 DTCs other than Leda and Teknaf DTCs serving these camps, and the settlements were in remotely located Ukhia and Teknaf sub-districts which are about 260 miles away from Dhaka, the capital city of Bangladesh.

Comment: Which DTCs were included in the study? I believe this was stated somewhere, but it was lost in the massive amount of background information included in the methods.

Response: Many thanks for your concerns. Teknaf and Leda DTCs were included in the study as those were serving FDMN and host population in Teknaf sub-district.

Comment: Who was invited to the survey? Suspected cases, lab confirmed cases or both?

Response: Our sincere thanks to you for your comments and suggestions. Laboratory confirmed cholera patients were the respondents or their parents who were administered a pre-tested questionnaire to collect relevant information.

Comment: Over what period were data collected? This was mentioned in the background and results, but it should be included in the methods.

Response: Thank you once again. Data were collected during September-December 2019. Necessary inclusions have been made in the Methods section. Line 196-198.

Comment: What language were the surveys conducted in and who exactly conducted the surveys? Were they trained?

Response: Our sincere thanks for raising this vital issue. We have responded in the Data collection section after revising that section. Line 216-227.

Reviewer #2: The article is sound in its methodology, objectives of the study are clearly

articulated. The study design is appropriate to address the stated objectives. The population is clearly described and appropriate. The correct statistical analysis was used to support the conclusion and the concerns about ethical or regulatory requirements were met.

Response: Thank you so much for the encouraging notes.

Reviewer #3: -The objectives of the study were very clear
-and the study design was appropriate to address the objectives of the study
- The population was clearly described and the sample was sufficient for the study objectives
-Correct statistical analysis was undertaken, but --------------------

Response: Thank you for your comments and suggestions. Necessary revisions have been made. Line 229-235.

Results

Reviewer #1: The results were well presented and matched the analysis plan. Appropriate statistical tests were run.

Response: Thank you for encouraging notes.

Comment: Tables 2 and 3 included some p values, but not others (which were mentioned in the narrative). I would suggest included all p-values in all tables.

Response: Many thanks for your observations and necessary revisions have been made in Tables. Line 262-264, Line 279-281.

Comment: What proportion of all camps served by these DTCs were affected? I see that 6 camps have data, but how many camps were served? ….camps were served. The outbreak was in a localized area …..camps out of ……camps as well as neighborhood host population.

Response: Thank you very much for your concern to know more about the scenario. Of the 34 camps, the reported outbreak was localized in 6 camps. Leda and Teknaf DTCs functioning in their neighborhood served those 6 camps along with cholera patients from nearby host communities.

Reviewer #2: Yes, the analysis was appropriately done. And the data were clearly presented. However, there is no graphical presentation of data. Adding graphical presentation or images would be nice.

Response: We have included the map of the Teknaf sub-district showing the location of 6 camps as well as Leda and Teknaf DTCs into our manuscript.

Reviewer #3: -A careful analysis was presented that matched the analysis plan
-The results were clear and tables were of sufficient quality

Response: Thank you for the encouraging notes.

Conclusions

Reviewer #1: The conclusions are supported by the data and the limitations are described. The importance of OVC was well established, but the other findings were less well discussed.

I am wondering about the significance of these results. What does this study tell us that other studies of cholera in refugee camps have not already established? How did these results inform policies and programs for this population?

Response: Thank you for your thoughtful concerns. Our results mentioned how preparedness as soon as emergency and crisis started for both preventive and control measures and response readiness, with active support from an ongoing effective disease surveillance system can help in addressing threats of cholera outbreaks. Disease surveillance continually generates essential epidemiologic data for effective strategy formulation as well as the implementation of effective control measures through inter-sectoral collaborations. A surveillance system is critical for early detection of disease outbreaks before any spread to other family members as well as individuals living in the neighborhood, unnecessarily costing lives and challenging the disease control efforts. Thus, our ongoing DTC-based diarrheal disease surveillance system with timely laboratory back-up and immediate reporting to all concerned agencies was noteworthy in this emergency and crisis setting. The surveillance system was involved not only in collecting reliable data since the inception of the DTC network but also in reporting immediately to help significantly in anticipating and detecting early potential cholera outbreaks. Findings from surveillance system guided intervention strategies that lead to the timely undertaking of preventive measures and the preparedness that included training of health care staff, opening of temporarily closed down Leda DTC, strengthening of the capacity of existing DTCs and outreach activities, as well as prepositioning of supplies and additional human resources. Other additional vital strategies undertaken immediately were strengthening of inter-sectoral collaboration, enhancing preventive and control measures (regular monitoring of the quality of drinking water sources at waterpoints and household level, sanitation and hygiene status) as well as augmentation of OCV campaigns. Efforts further emphasized preparedness for surges and vigilance of cholera patients which is the priority undertakings of the Health Systems of Government of Bangladesh (the host country) because of existing threats of cholera in both the host and displaced populations in emergency and crisis settings. Additionally, mass OCV campaigns for FDMN as well as the host population can reduce the threats of cholera in both the host and FDMN.

Structurally, I found the authors jumped around a bit too much. They started by discussing general findings, then specific issues around OVC. Then they turned to a focus on water, sanitation, and ORS, before returning to OVC in Sudan. Why not include all discussions of OVC together in one place? I found this difficult to follow and parse out the main points.

Response: Thank you for your comments and suggestions. The present study observed that 94% of the host community individuals and two-third of the FDMN with laboratory-confirmed cholera were not exposed to OCV before getting hospitalized with AWD. A recent experience from Bangladesh and India indicated that the protective efficacy of Shanchol OCV (produced in India) among those more than five years against cholera is 53-65%. The study mentioned the positive

role of OCV as a pre-emptive measure in endemic settings, in natural or man-made disasters even in disruptive situations with a breakdown of WASH and absence of other disease control and public health measures. WHO and Global Task Force for Cholera Control (GTFCC) recommend that a comprehensive multi-sectoral involvement is important for the successful elimination of cholera. Mass OCV campaigns with high coverage are feasible even after the arrival of a large number of displaced populations in a distressed state in resource-poor settings like Bangladesh. According to another study, OCV induced optimal immune responses in FDMN adults and children which were similar to that observed in Bangladesh's population of diverse age groups or individuals living in other cholera endemic countries. In Sudan among the displaced populations, the risks for cholera were considerably higher among children less than five years living in refugee camps. A Cochrane review indicated significantly lower protective efficiency of OCV in under-five children compared to children who are older than them as well as adults. Vigilance for cholera individuals as well as preparedness for prevention and mitigation measures for surges and mass OCV campaigns for FDMN as well as host population can reduce the threats of cholera in both the host and FDMN. Line 384-395.

Comments: In the section on ORS use, I think the authors miss a big point. They highlight the role that limited access to ORS plays, but then they explain that the refugee population might not be motivated to use ORS. What about knowledge? A big factor limiting people's use of ORS is their knowledge of when and how to use this vital tool. This appears to be blaming the victim rather than focusing on the limitations of the system in which they live. Further, no mention of water treatment is made, which in refugee camps is a vital tool to providing safe water when wells are inadequate.

Response: Thank you for your kind comments and suggestions. An important factor limiting people's use of ORS is their knowledge of when and how to use this vital tool. Major limitations of outreach activities in this emergency and crisis scenario may include less promotion and access to ORS packets at the household or community level in settlements, because of less organized outreach activities. Additionally, lack of appropriate health education measures to enhance FDMN's knowledge about ORS use particularly their awareness on when to start, how to prepare, how much to be taken, and how long ORS to be continued. All these more effective attempts may motivate FDMNs to enhance their appropriate use of ORS at the household level before coming to DTCs. Treatment of water is a vital tool for providing safe water when tube wells are inadequate in meeting the needs of the displaced population in emergency and crisis settings. Line 349-356.

Reviewer #2: Yes the conclusions are on the basis of study findings. The discussions are adequate and well argued with evidence.
The study is unique in two ways : one, it is about Cholera outbreak which is a public health emergency. Two, the population is forcibly displaced vulnerable group. The underlying cross-cutting issues are well discussed.

Response: Many thanks for your appreciation.

Reviewer #3: -The conclusions are supported by the data and limitations clearly described.

-Authors have discussed how the study health public health understanding AWDs in humanitarian crisis as well as the public health relevance of the study.

Response: We are extremely happy to know your very encouraging comments.

Reviewer #1: One issue I had with this manuscript was the use of the term case rather than people or patients. Towards the end of the results the terms patient or female/child case were used, which is an improvement because it humanizes this population. This is already a highly vulnerable population and reducing them down to a non-human cases is unnecessary and potentially harmful. I would suggest the more humanistic term and to standardize the term throughout.

Response: Thanks a lot for your thoughtful observations and valuable suggestions. Accordingly, we have made all needed revisions in the text of the manuscript.

There were other minor grammatical and editorial issues I noted throughout (see attached) Finally, see my previous comments about the organization of the paper. Much of the methods I feel could be moved to the background and again to the discussion. That way the results are framed around the beginning of the cholera outbreak and how the group initially responded, and then how they used these surveillance systems and results to inform programming and policy.

Response: Our sincere thanks for sharing very vital suggestions. Necessary revisions have been made.

Reviewer #2: Minor revision

Reviewer #3: Minor revisions
- Authors need to include ethical approval number in the ethical statement. They also need to bring the ethical statement at the start of the methods section.

Response: Many thanks. We included the ethical approval number in the ethical statement. That has been moved to the start of Methods section. Necessary revisions have been made. Line 184-192.

Major revision
- A map of the study setting showing camps were patients originated and locations of the treatment centers would highly enrich this study. Please see my comments in the paper.

Response: Thank you for very helpful comments and suggestions. We have included a map as Figure 1 that describes location of the DTCs and camps and their neighborhood from where cholera patients sought care.

Summary and General Comment

Use this section to provide overall comments, discuss strengths/weaknesses of the study, novelty, significance, general execution and scholarship. You may also include additional comments for the author, including concerns about dual publication, research ethics, or publication ethics. If requesting major revision, please articulate the new experiments that are needed.

Reviewer #1: Overall, this paper presents novel data about a cholera outbreak amongst Myanmar refugees in Bangladesh, highlighting the important role that OVC plays in preventing disease. It also discusses the demographic makeup and health seeking behaviors of this population. However, I am left wondering what the significance is. How does this advance the literature of cholera in refugee populations in general, and specifically in Bangladesh? How did/could these results inform policy or programming?

Comments: Many thanks for sharing your concerns. Immediately after the arrival of a large number of displaced population, apprehending the threats of cholera outbreaks, the Government of Bangladesh as lead, with technical support from icddr,b collaborating with international agencies, and international and national NGOs under the wider platform of Health Sector, undertook a massive oral cholera vaccination (OCV) campaign as a pre-emptive measure to alleviate threats of cholera outbreaks. Despite that mass OCV campaign, threats of cholera outbreaks among FDMN were existing due to new arrivals of the displaced population with compromised host susceptibility, frequent visits to settlements by Bangladesh nationals living in the neighboring community without exposure to OCV, and the decay of vaccine immunity in OCV recipients as well as an increasing number of cohort children without any exposure to OCV. Preparedness for preventive and control measures to combat surges and vigilance for people with cholera was the most important public health priority because of prevailing threats of cholera in both the host and displaced population. Between September and December 2019, there were 147 culture-confirmed cholera patients presented and were subsequently hospitalized with acute dehydrating diarrhea episodes in Leda and Teknaf DTCs. That did happen after two years of successful mass OCV campaigns. Among these cholera cases, infants and overall children <5 years old presented more frequently to the DTCs (functioning to treat FDMN living in settlements as well as host community individuals) compared to cholera children presenting to Dhaka Hospital from Dhaka city and its suburbs. However, for individuals aged 15 years and older, more cholera patients reported to Dhaka Hospital as opposed to cholera patients living in settlements and seeking care from DTCs. Significantly more female cholera patients visited DTCs as opposed to female cholera patients presenting to Dhaka Hospital. A recent experience from Bangladesh and India indicated that the protective efficacy of Shanchol OCV (produced in India) among those more than five years against cholera is 53-65%. The study mentioned the positive role of OCV as a pre-emptive measure in endemic settings, in natural or man-made disasters even in disruptive situations with a breakdown of WASH and absence of other disease control and public health measures. WHO and Global Task Force for Cholera Control (GTFCC) recommend that a comprehensive multi-sectoral involvement is important for the successful elimination of cholera. Mass OCV campaigns with high coverage are feasible even after the arrival of a large number of displaced populations in a distressed state in resource-poor settings like

Bangladesh. According to another study, OCV induced optimal immune responses in FDMN adults and children which were similar to that observed in Bangladesh's population of diverse age groups or individuals living in other cholera endemic countries. In Sudan among the displaced populations, the risks for cholera were considerably higher among children less than five years living in refugee camps. A Cochrane review indicated significantly lower protective efficiency of OCV in under-five children compared to children who are older than them as well as adults.

Based on these observations we would conclude that policymakers may plan continued vigilance for cholera individuals as well as preparedness for prevention and mitigation measures for surges particularly that of cholera and mass OCV campaigns for FDMN as well as host population which can reduce the threats of cholera in both the host and FDMN.

Reviewer #2: Despite being a well-planned study there are few places to revise in the manuscript. Line 122-125 : might need revision. As these statements praise the work of authors-affiliated organizations.

Response: Many thanks for your valuable observations. Accordingly, we have made necessary revisions. Line 121-123.

Comment: Line: 157-162 : The meeting in person by public health officials (DG) with agencies might be a procedure that does not need to be recalled in scientific article. It is well established that coordination is vital.

Thanks so much once again for pointing out that issue. We have made necessary revisions. Line 145-160.

Reviewer #3: This study is very relevant to informing prevention and control interventions during humanitarian crises context. It is a significant study in the field of public health emergencies and contains needed data in moving the field forward.

Response: Many thanks for encouraging notes.

Figure 1

Please provide us with a direct link to the base layer of the map used in [Figure 1] and ensure this location is also included in the figure legend. Please note that, because all PLOS articles are published under a CC BY license (creativecommons.org/licenses/by/4.0/), we cannot publish proprietary maps such as Google Maps, Mapquest or other copyrighted maps. If your map was obtained from a copyrighted source please amend the figure so that the base map used is from an openly available source. Alternatively, please provide explicit written permission from the copyright holder granting you the right to publish the material under a CC-BY 4.0 license.

Please note that only the following CC BY licences are compatible with PLOS licence: CC BY 4.0, CC BY 2.0 and CC BY 3.0, meanwhile such licences as CC BY-ND 3.0 and others are not

compatible due to additional restrictions. If you are unsure whether you can use a map or not, please do reach out and we will be able to help you.

Response: This graph has been made based on the number of cholera cases (combined of settlement and host community) by using the R language with tmap package. So there has no issue of copyright. For more clarification, we have shared our working R script, data, and output (attached zip file) for your kind review.

Click here to access/download
Revised Article with Changes Highlighted
Plos NTD_Coxs_Bazar_ASGF. revised.20.5.2021.docx
