# Supplementary material for: Cholera outbreak in Forcibly Displaced Myanmar National (FDMN) from a small population segment in Cox’s Bazar, Bangladesh, 2019
Source: PLoS Negl Trop Dis. 2021 Sep 22;15(9):e0009618. doi: 10.1371/journal.pntd.0009618 (PMC8457470; doi:10.1371/journal.pntd.0009618)
Supplement: Attachment — Submitted filename: Response to Reviewers comment_Plos NTD.docx [file pntd.0009618.s006.docx]

**Point-by-point responses to reviewer’s comment:**

**Methods**

**Reviewer #1**: Overall, the objectives were clearly stated in the background section of the manuscript. However, I found the objectives, as written in the abstract, to be too long and comprehensive to follow. I would suggest shortening the objectives to a simple statement about collecting cholera epidemiology in a sample of refugees and host country nationals.

**Response:** Thank you for your kind suggestions. The objectives in the abstract have been shortened to a simple statement. Line 27-30.

**Comment:** The study design was appropriate for these objectives. I found the methods section to be a little confusing. The first half (lines 139-185) of the methods appeared to be more of a background about the outbreak than the specific methods of the study. I strongly suggest that portions of that section be moved to the background, with others (how did they respond to the outbreak) moved to the conclusion. That way the results are framed around those two pieces of context.

**Response:** Many thanks for your valuable suggestions. Suggested portions of the Methods section have been moved to the Introduction section. As also suggested, other portions have been moved to the Discussion section. Line 128-160.

**Comment:** Further, this first half of the methods had a sub-header (setting and study population), but the rest of the methods section did not. I suggest including additional headers to help guide the reader and follow what to me was a very complex section of the paper. These could include descriptions of the surveillance systems, survey and lab methods, statistical methods, and ethical review.

**Response:** Our heartfelt thanks for the valuable comments and suggestions. We have followed your suggestions and included other sub-headings like (i) Stool sample collection, rapid diagnostic testing, and laboratory methods, (ii) Data collection, and (iii) Statistical Analysis. Line 194-235

**Comment:** I have some specific comments/questions:
-Why was Dhaka used as a comparison sample rather than a nearby clinic? One would expect patients in the high-density urban center of Bangladesh to be very different from refugees living in camps. I applaud the inclusion of host nationals living in nearby settlements, but do not understand the inclusion of this comparator group.

**Response:** Thank you once again for your thoughtful concerns. Written below are our explanations for using Dhaka as a comparison sample. We are delighted to have your appreciation for the inclusion of host community in our analysis.

There was an absence of comparable diarrhea treatment facilities in the settlements which not only providing quality care but also routinely examining stool specimens for diarrheagenic enteric organisms following standard laboratory methods. We needed data for comparison of presenting clinical and demographic features of hospitalized cholera patients (such as age, sex, duration of diarrhea, watery stool, dehydration status, and pre-packaged ORS use at home before reporting) from Leda and Teknaf DTCs with that of a facility that has a track record of diarrheal disease surveillance system and treating hospitalized cholera patients who are seeking care from such a facility that does not charge for providing quality care mostly from those attending from poor socio-economic contexts, remains open round-the-clock, and can efficiently handle sudden upsurges of patients including individuals with cholera presenting often in a dehydrated state in relatively large number and the facility has back-up laboratory support for routine fecal specimen examinations following standard methods for detection and characterization of causative enteric organisms including *V. cholerae.* Line 386-397.

**Comment:** Some additional context on the number of camps, number of DTCs serving those camps, and how far they are from Dhaka would be helpful. As someone with limited knowledge of Bangladesh, this context would help me understand the context of the results.

**Response:** Thank you. There were 34 camps, 8 DTCs other than Leda and Teknaf DTCs serving these camps, and the settlements were in remotely located Ukhia and Teknaf sub-districts which are about 260 miles away from Dhaka, the capital city of Bangladesh.

**Comment:** Which DTCs were included in the study? I believe this was stated somewhere, but it was lost in the massive amount of background information included in the methods.

**Response:** Many thanks for your concerns. Teknaf and Leda DTCs were included in the study as those were serving FDMN and host population in Teknaf sub-district.

**Comment:** Who was invited to the survey? Suspected cases, lab confirmed cases or both?

**Response:** Our sincere thanks to you for your comments and suggestions. Laboratory confirmed cholera patients were the respondents or their parents who were administered a pre-tested questionnaire to collect relevant information.

**Comment:** Over what period were data collected? This was mentioned in the background and results, but it should be included in the methods.

**Response:** Thank you once again. Data were collected during September-December 2019. Necessary inclusions have been made in the Methods section. Line 196-198.

**Comment:** What language were the surveys conducted in and who exactly conducted the surveys? Were they trained?

**Response:** Our sincere thanks for raising this vital issue. We have responded in the Data collection section after revising that section. Line 216-227.

**Reviewer #2:** The article is sound in its methodology, objectives of the study are clearly articulated. The study design is appropriate to address the stated objectives. The population is clearly described and appropriate. The correct statistical analysis was used to support the conclusion and the concerns about ethical or regulatory requirements were met.

**Response:** Thank you so much for the encouraging notes.

**Reviewer #3:** -The objectives of the study were very clear
-and the study design was appropriate to address the objectives of the study
- The population was clearly described and the sample was sufficient for the study objectives
-Correct statistical analysis was undertaken, but --------------------

**Response:** Thank you for your comments and suggestions. Necessary revisions have been made. Line 229-235.

**Results**

**Reviewer #1**: The results were well presented and matched the analysis plan. Appropriate statistical tests were run.

**Response:** Thank you for encouraging notes.

**Comment:** Tables 2 and 3 included some p values, but not others (which were mentioned in the narrative). I would suggest included all p-values in all tables.

**Response:** Many thanks for your observations and necessary revisions have been made in Tables. Line 262-264, Line 279-281.

**Comment:** What proportion of all camps served by these DTCs were affected? I see that 6 camps have data, but how many camps were served? ….camps were served. The outbreak was in a localized area …..camps out of ……camps as well as neighborhood host population.

**Response:** Thank you very much for your concern to know more about the scenario. Of the 34 camps, the reported outbreak was localized in 6 camps. Leda and Teknaf DTCs functioning in their neighborhood served those 6 camps along with cholera patients from nearby host communities.

**Reviewer #2:** Yes, the analysis was appropriately done. And the data were clearly presented. However, there is no graphical presentation of data. Adding graphical presentation or images would be nice.

**Response:** We have included the map of the Teknaf sub-district showing the location of 6 camps as well as Leda and Teknaf DTCs into our manuscript.

**Reviewer #3:** -A careful analysis was presented that matched the analysis plan
-The results were clear and tables were of sufficient quality

**Response:** Thank you for the encouraging notes.

**Conclusions**

**Reviewer #1**: The conclusions are supported by the data and the limitations are described. The importance of OVC was well established, but the other findings were less well discussed.

I am wondering about the significance of these results. What does this study tell us that other studies of cholera in refugee camps have not already established? How did these results inform policies and programs for this population?

**Response:** Thank you for your thoughtful concerns. Our results mentioned how preparedness as soon as emergency and crisis started for both preventive and control measures and response readiness, with active support from an ongoing effective disease surveillance system can help in addressing threats of cholera outbreaks. Disease surveillance continually generates essential epidemiologic data for effective strategy formulation as well as the implementation of effective control measures through inter-sectoral collaborations. A surveillance system is critical for early detection of disease outbreaks before any spread to other family members as well as individuals living in the neighborhood, unnecessarily costing lives and challenging the disease control efforts. Thus, our ongoing DTC-based diarrheal disease surveillance system with timely laboratory back-up and immediate reporting to all concerned agencies was noteworthy in this emergency and crisis setting. The surveillance system was involved not only in collecting reliable data since the inception of the DTC network but also in reporting immediately to help significantly in anticipating and detecting early potential cholera outbreaks. Findings from surveillance system guided intervention strategies that lead to the timely undertaking of preventive measures and the preparedness that included training of health care staff, opening of temporarily closed down Leda DTC, strengthening of the capacity of existing DTCs and outreach activities, as well as prepositioning of supplies and additional human resources. Other additional vital strategies undertaken immediately were strengthening of inter-sectoral collaboration, enhancing preventive and control measures (regular monitoring of the quality of drinking water sources at waterpoints and household level, sanitation and hygiene status) as well as augmentation of OCV campaigns. Efforts further emphasized preparedness for surges and vigilance of cholera patients which is the priority undertakings of the Health Systems of Government of Bangladesh (the host country) because of existing threats of cholera in both the host and displaced populations in emergency and crisis settings. Additionally, mass OCV campaigns for FDMN as well as the host population can reduce the threats of cholera in both the host and FDMN.

Structurally, I found the authors jumped around a bit too much. They started by discussing general findings, then specific issues around OVC. Then they turned to a focus on water, sanitation, and ORS, before returning to OVC in Sudan. Why not include all discussions of OVC together in one place? I found this difficult to follow and parse out the main points.

**Response**: Thank you for your comments and suggestions. The present study observed that 94% of the host community individuals and two-third of the FDMN with laboratory-confirmed cholera were not exposed to OCV before getting hospitalized with AWD. A recent experience from Bangladesh and India indicated that the protective efficacy of Shanchol OCV (produced in India) among those more than five years against cholera is 53-65%. The study mentioned the positive role of OCV as a pre-emptive measure in endemic settings, in natural or man-made disasters even in disruptive situations with a breakdown of WASH and absence of other disease control and public health measures. WHO and Global Task Force for Cholera Control (GTFCC) recommend that a comprehensive multi-sectoral involvement is important for the successful elimination of cholera. Mass OCV campaigns with high coverage are feasible even after the arrival of a large number of displaced populations in a distressed state in resource-poor settings like Bangladesh.  According to another study, OCV induced optimal immune responses in FDMN adults and children which were similar to that observed in Bangladesh's population of diverse age groups or individuals living in other cholera endemic countries. In Sudan among the displaced populations, the risks for cholera were considerably higher among children less than five years living in refugee camps. A Cochrane review indicated significantly lower protective efficiency of OCV in under-five children compared to children who are older than them as well as adults. Vigilance for cholera individuals as well as preparedness for prevention and mitigation measures for surges and mass OCV campaigns for FDMN as well as host population can reduce the threats of cholera in both the host and FDMN. Line 384-395.

**Comments:** In the section on ORS use, I think the authors miss a big point. They highlight the role that limited access to ORS plays, but then they explain that the refugee population might not be motivated to use ORS. What about knowledge? A big factor limiting people's use of ORS is their knowledge of when and how to use this vital tool. This appears to be blaming the victim rather than focusing on the limitations of the system in which they live. Further, no mention of water treatment is made, which in refugee camps is a vital tool to providing safe water when wells are inadequate.

**Response:** Thank you for your kind comments and suggestions. An important factor limiting people's use of ORS is their knowledge of when and how to use this vital tool. Major limitations of outreach activities in this emergency and crisis scenario may include less promotion and access to ORS packets at the household or community level in settlements, because of less organized outreach activities. Additionally, lack of appropriate health education measures to enhance FDMN’s knowledge about ORS use particularly their awareness on when to start, how to prepare, how much to be taken, and how long ORS to be continued. All these more effective attempts may motivate FDMNs to enhance their appropriate use of ORS at the household level before coming to DTCs. Treatment of water is a vital tool for providing safe water when tube wells are inadequate in meeting the needs of the displaced population in emergency and crisis settings. Line 349-356.

**Reviewer #2:** Yes the conclusions are on the basis of study findings. The discussions are adequate and well argued with evidence.
The study is unique in two ways : one, it is about Cholera outbreak which is a public health emergency. Two, the population is forcibly displaced vulnerable group. The underlying cross-cutting issues are well discussed.

**Response:** Many thanks for your appreciation.

**Reviewer #3:** -The conclusions are supported by the data and limitations clearly described.
-Authors have discussed how the study health public health understanding AWDs in humanitarian crisis as well as the public health relevance of the study.

**Response:** We are extremely happy to know your very encouraging comments.


**Reviewer #1:** One issue I had with this manuscript was the use of the term case rather than people or patients. Towards the end of the results the terms patient or female/child case were used, which is an improvement because it humanizes this population. This is already a highly vulnerable population and reducing them down to a non-human cases is unnecessary and potentially harmful. I would suggest the more humanistic term and to standardize the term throughout.

**Response:** Thanks a lot for your thoughtful observations and valuable suggestions. Accordingly, we have made all needed revisions in the text of the manuscript.

There were other minor grammatical and editorial issues I noted throughout (see attached)
Finally, see my previous comments about the organization of the paper. Much of the methods I feel could be moved to the background and again to the discussion. That way the results are framed around the beginning of the cholera outbreak and how the group initially responded, and then how they used these surveillance systems and results to inform programming and policy.

**Response:** Our sincere thanks for sharing very vital suggestions. Necessary revisions have been made.

**Reviewer #2**: Minor revision

**Reviewer #3**: Minor revisions
- Authors need to include ethical approval number in the ethical statement. They also need to bring the ethical statement at the start of the methods section.

**Response:** Many thanks. We included the ethical approval number in the ethical statement. That has been moved to the start of Methods section. Necessary revisions have been made. Line 184-192.

**Major revision**
- A map of the study setting showing camps were patients originated and locations of the treatment centers would highly enrich this study. Please see my comments in the paper.

**Response**: Thank you for very helpful comments and suggestions. We have included a map as Figure 1 that describes location of the DTCs and camps and their neighborhood from where cholera patients sought care.

**Summary and General Comment**

Use this section to provide overall comments, discuss strengths/weaknesses of the study, novelty, significance, general execution and scholarship. You may also include additional comments for the author, including concerns about dual publication, research ethics, or publication ethics. If requesting major revision, please articulate the new experiments that are needed.

**Reviewer #1:** Overall, this paper presents novel data about a cholera outbreak amongst Myanmar refugees in Bangladesh, highlighting the important role that OVC plays in preventing disease. It also discusses the demographic makeup and health seeking behaviors of this population. However, I am left wondering what the significance is. How does this advance the literature of cholera in refugee populations in general, and specifically in Bangladesh? How did/could these results inform policy or programming?

**Comments:** Many thanks for sharing your concerns. Immediately after the arrival of a large number of displaced population, apprehending the threats of cholera outbreaks, the Government of Bangladesh as lead, with technical support from icddr,b collaborating with international agencies, and international and national NGOs under the wider platform of Health Sector, undertook a massive oral cholera vaccination (OCV) campaign as a pre-emptive measure to alleviate threats of cholera outbreaks. Despite that mass OCV campaign, threats of cholera outbreaks among FDMN were existing due to new arrivals of the displaced population with compromised host susceptibility, frequent visits to settlements by Bangladesh nationals living in the neighboring community without exposure to OCV, and the decay of vaccine immunity in OCV recipients as well as an increasing number of cohort children without any exposure to OCV. Preparedness for preventive and control measures to combat surges and vigilance for people with cholera was the most important public health priority because of prevailing threats of cholera in both the host and displaced population. Between September and December 2019, there were 147 culture-confirmed cholera patients presented and were subsequently hospitalized with acute dehydrating diarrhea episodes in Leda and Teknaf DTCs. That did happen after two years of successful mass OCV campaigns. Among these cholera cases, infants and overall children <5 years old presented more frequently to the DTCs (functioning to treat FDMN living in settlements as well as host community individuals) compared to cholera children presenting to Dhaka Hospital from Dhaka city and its suburbs. However, for individuals aged 15 years and older, more cholera patients reported to Dhaka Hospital as opposed to cholera patients living in settlements and seeking care from DTCs. Significantly more female cholera patients visited DTCs as opposed to female cholera patients presenting to Dhaka Hospital. A recent experience from Bangladesh and India indicated that the protective efficacy of Shanchol OCV (produced in India) among those more than five years against cholera is 53-65%. The study mentioned the positive role of OCV as a pre-emptive measure in endemic settings, in natural or man-made disasters even in disruptive situations with a breakdown of WASH and absence of other disease control and public health measures. WHO and Global Task Force for Cholera Control (GTFCC) recommend that a comprehensive multi-sectoral involvement is important for the successful elimination of cholera. Mass OCV campaigns with high coverage are feasible even after the arrival of a large number of displaced populations in a distressed state in resource-poor settings like Bangladesh.  According to another study, OCV induced optimal immune responses in FDMN adults and children which were similar to that observed in Bangladesh's population of diverse age groups or individuals living in other cholera endemic countries. In Sudan among the displaced populations, the risks for cholera were considerably higher among children less than five years living in refugee camps. A Cochrane review indicated significantly lower protective efficiency of OCV in under-five children compared to children who are older than them as well as adults.

Based on these observations we would conclude that policymakers may plan continued vigilance for cholera individuals as well as preparedness for prevention and mitigation measures for surges particularly that of cholera and mass OCV campaigns for FDMN as well as host population which can reduce the threats of cholera in both the host and FDMN.

**Reviewer #2:** Despite being a well-planned study there are few places to revise in the manuscript. Line 122-125 : might need revision. As these statements praise the work of authors-affiliated organizations.

**Response:** Many thanks for your valuable observations. Accordingly, we have made necessary revisions. Line 121-123.

**Comment:** Line: 157-162 : The meeting in person by public health officials (DG) with agencies might be a procedure that does not need to be recalled in scientific article. It is well established that coordination is vital.

Thanks so much once again for pointing out that issue. We have made necessary revisions. Line 145-160.
**Reviewer #3:** This study is very relevant to informing prevention and control interventions during humanitarian crises context. It is a significant study in the field of public health emergencies and contains needed data in moving the field forward.

**Response:** Many thanks for encouraging notes.

**Figure 1**

Please provide us with a direct link to the base layer of the map used in [Figure 1] and ensure this location is also included in the figure legend. Please note that, because all PLOS articles are published under a CC BY license (creativecommons.org/licenses/by/4.0/), we cannot publish proprietary maps such as Google Maps, Mapquest or other copyrighted maps. If your map was obtained from a copyrighted source please amend the figure so that the base map used is from an openly available source. Alternatively, please provide explicit written permission from the copyright holder granting you the right to publish the material under a CC-BY 4.0 license.

Please note that only the following CC BY licences are compatible with PLOS licence: CC BY 4.0, CC BY 2.0 and CC BY 3.0, meanwhile such licences as CC BY-ND 3.0 and others are not compatible due to additional restrictions. If you are unsure whether you can use a map or not, please do reach out and we will be able to help you.

**Response:** This graph has been made based on the number of cholera cases (combined of settlement and host community) by using the R language with tmap package. So there has no issue of copyright. For more clarification, we have shared our working R script, data, and output (attached zip file) for your kind review.
